# A large deletion conferring pale green leaves of maize

**DOI:** 10.1186/s12870-023-04360-2

**Published:** 2023-07-14

**Authors:** Guoqi Yao, Hua Zhang, Bingying Leng, Bing Cao, Juan Shan, Zhenwei Yan, Haiying Guan, Wen Cheng, Xia Liu, Chunhua Mu

**Affiliations:** 1 Maize Research Institute, Shandong Academy of Agricultural Sciences, Jinan, 250100 China; 2 Key Laboratory of Biology and Genetic Improvement of Maize in Northern Yellow-Huai River Plain, Ministry of Agriculture, Jinan, 250100 China; 3 National Engineering Laboratory of Wheat and Maize, Jinan, 250100 China; 4 National Maize Improvement Sub-Center, Jinan, 250100 China; 5 College of Life Sciences, Shandong Normal University, Jinan, 250014 China

**Keywords:** Maize, Pgl, Map based cloning, Large deletion

## Abstract

**Background:**

The structural basis of chloroplast and the regulation of chloroplast biogenesis remain largely unknown in maize. Gene mutations in these pathways have been linked to the abnormal leaf color phenotype observed in some mutants. Large scale structure variants (SVs) are crucial for genome evolution, but few validated SVs have been reported in maize and little is known about their functions though they are abundant in maize genomes.

**Results:**

In this research, a spontaneous maize mutant, *pale green leaf-shandong* (*pgl-sd*), was studied. Genetic analysis showed that the phenotype of pale green leaf was controlled by a recessive Mendel factor mapped to a 156.8-kb interval on the chromosome 1 delineated by molecular markers gy546 and gy548. There were 7 annotated genes in this interval. Reverse transcription quantitative PCR analysis, SV prediction, and de novo assembly of *pgl-sd* genome revealed that a 137.8-kb deletion, which was verified by Sanger sequencing, might cause the *pgl-sd* phenotype. This deletion contained 5 annotated genes, three of which, including *Zm00001eb031870, Zm00001eb031890* and *Zm00001eb031900,* were possibly related to the chloroplast development*. Zm00001eb031870,* encoding a Degradation of Periplasmic Proteins (Deg) homolog, and *Zm00001eb031900*, putatively encoding a plastid pyruvate dehydrogenase complex E1 component subunit beta (ptPDC-E1-β), might be the major causative genes for the *pgl-sd* mutant phenotype*.* Plastid Degs play roles in protecting the vital photosynthetic machinery and ptPDCs provide acetyl-CoA and NADH for fatty acid biosynthesis in plastids, which were different from functions of other isolated maize leaf color associated genes. The other two genes in the deletion were possibly associated with DNA repair and disease resistance, respectively. The *pgl-sd* mutation decreased contents of chlorophyll a, chlorophyll b, carotenoids by 37.2%, 22.1%, and 59.8%, respectively, and led to abnormal chloroplast. RNA-seq revealed that the transcription of several other genes involved in the structure and function of chloroplast was affected in the mutant.

**Conclusions:**

It was identified that a 137.8-kb deletion causes the *pgl-sd* phenotype. Three genes in this deletion were possibly related to the chloroplast development, which may play roles different from that of other isolated maize leaf color associated genes.

**Supplementary Information:**

The online version contains supplementary material available at 10.1186/s12870-023-04360-2.

## Background

Mutants with the phenotype of abnormal leaf color have been reported frequently, most of which can be grouped as *albina*, *xantha*, *alboviridis*, *viridis*, and *girina* [15]. In general, these phenotypes were related to mutation of genes in the pathway of chlorophyll synthesis and degradation or genes directly involved in the chloroplast biogenesis. For example, green genes identified in *Arabidopsis* [1], and rice genes *Ygl1* [51], *Ygl2* [8], *Ygl3* [46], *Ygl7* [10] and *Ygl80* [45] are responsible for chlorophyll metabolism, while *Arabidopsis* genes *Apg1* ( [37], *Cao* [23]*, Egy1* [7] and *Var3* [36], and rice genes *Ygl138(T)* [55] and *Vyl* [11] encode proteins for the development of chloroplast. In maize, more than 200 genes/Quantitative Trait Loci (QTL) associated with leaf color have been recorded in maizeGDB database [54], including 8 isolated genes, *Elm1*, *Elm2*, *Chr.1-ClpP5*, *Oy1*, *Oy2, Vyl*, *Ygl-1*, and *Zb7*. *Elm1* encodes a phytochromobilin synthase. *elm1,* a mutant of single base transition of *Elm1*, was deficient in phytochrome response and had a lower content of chlorophyll than wild plants under white light condition [39, 40]. *Elm2,* encoding a heme oxygenase, was also involved in phytochrome biosynthesis. *elm2* with a 21-bp deletion in *Elm2* showed yellow green leaves [42]. *Vyl* and *Chr.1-ClpP5* were a pair of *ClpP5* homologs. a 141-bp insertion in *Vyl* led to virescent yellow-like leaves [52]. *Oy1* encodes the subunit I of magnesium chelatase in the chlorophyll biosynthesis pathway*.* Semi-dominant *oy1* was a chlorophyll deficient mutant [41]. *Oy2* possibly encodes chelatase subunit D and a point mutation of this gene likely conferred the yellow leaves of maize [54]. *Ygl-1* possibly encodes a cpSRP43 protein required to target light-harvesting chlorophyll protein to thylakoid membrane. *ygl-1,* a mutant of single nucleotide deletion of *Ygl-1,* showed yellow-green leaves [14]. Zb7 is an IPP and DMAPP synthase involving in isoprenoid synthesis. A single nucleotide alteration of *Zb7* decreased the biosynthesis of chlorophyll and carotenoid leading to transverse yellow-green leaf phenotype [32].

Though most of identified genomic variants were single nucleotide polymorphisms (SNPs) or Indel polymorphisms (IDPs), large scale structure variants (SVs), classified as genome rearrangements typically larger than 100 bp [18], were also abundant in the crop genome as revealed in maize by genome sequence projects [28, 43]. SVs contributed to the adaptation of crops to environments and the variation source for breeding. For example, a 38.3-kb deletion in rice harbored *grain number, plant height and heading date7* (*Ghd7*) [53], and a 254-kb deletion in soybean was associated with a low level of palmitic acid of seeds [13]. However, few SVs in maize have been verified with experiments and little is known about their biological functions. A 147-kb deletion identified in maize containing maize *wall-associated kinase* (*ZmWAK*) resulted in susceptibility to the fungal disease head smut [58]. Han et al. [16] reported that a 5.16-Mb deletion led to a set of phenotypic abnormities, including reduced plant height, increased stomatal density, and rapid water losing in maize.

In this research, a natural maize mutant with pale green leaf, pale green leaf-shandong (*pgl-sd*), was studied. Map-based cloning revealed that a 137.8-kb deletion containing 5 genes in chromosome 1 of *pgl-sd* results in its abnormal leaf color.

## Results

### Inheritance of leaf color of *pgl-sd*

**Fig. 1:**
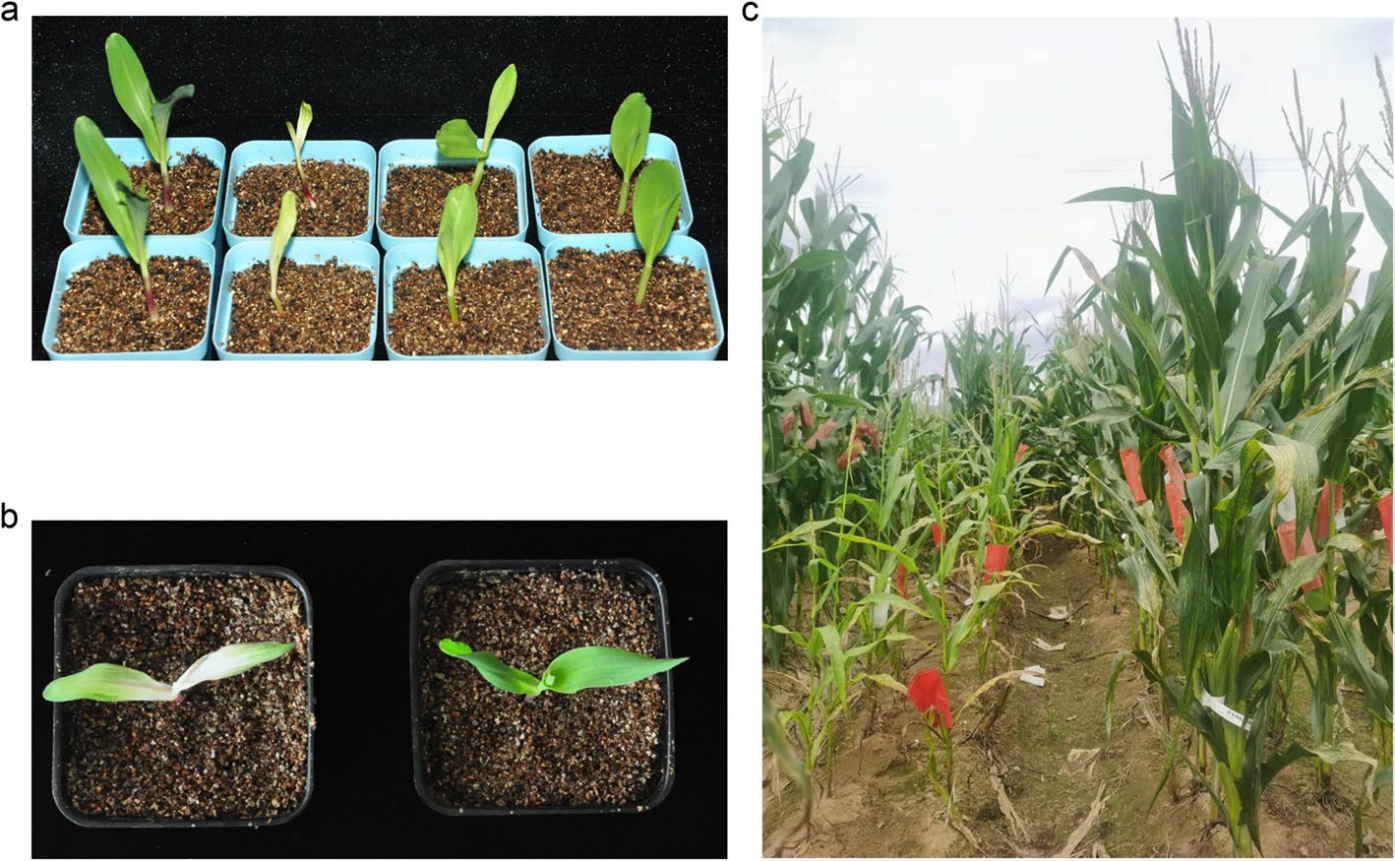
The phenotype of *pgl-sd*. **a** seedlings of B73 (column 1), *pgl-sd* (column 2), Zheng58 (column 3), Qi319 (column 4). **b** seedlings of a mutant (left) and a wild plant (right) from B73/*2*pgl-sd* BC_1_ population. **c** adult plants of *pgl-sd* (left) and Qi319 (right) growing in the field

The mutant *pgl-sd* had leaves with nearly white color at the base and pale green at the tip at seedling stages, but it exhibited pale green leaves at later growth stages (Fig. 1), a phenotype similar to that of *B73vyl* [52]. The mutant was shorter and had later anthesis and silking date relative to the three wild inbred lines, B73, Zheng58 and Qi319, but could grow to maturity and produce seeds.

The F_1_ progeny of the crosses between *pgl-sd* with B73, Zheng58 or Qi319 showed green leaves and grew normally as the wild parents. Among a BC_1_ population of the cross between *pgl-sd* and B73, 41 of 98 individuals exhibited *pgl-sd* mutant phenotype. The ratio of the mutant to the wild-type was in agreement with a 1:1 segregation ratio (*X*^2^_(1:1)_ = 2.61, *P* = 0.11). In a Zheng58/*pgl-sd* F_2_ population (Population 1), 26 mutant plants and 69 wild were observed, respectively (*X*^2^_(1:3)_ = 0.28, and *P* = 0.59). These data suggested that the *pgl-sd* mutant phenotype was probably determined by a single recessive gene. These two populations were used for the initial mapping of *pgl-sd.*

At the fine mapping stage, we further investigated leaf color of 3635 individuals from a Zheng58/*pgl-sd* F_2_ population and 2521 plants from a Qi319/*pgl-sd* F_2_ population, respectively. A total of 598 plants in the former population and 405 in the latter expressed the mutant phenotype, respectively. The mutant frequencies of both populations were approximately 16%, which were significantly lower than expected 25%. In the BC_1_ population mentioned above, the mutant frequency was not significantly different from 50%, but it was closer to 40% (*P* = 0.71), which was consistent with the segregation ratio of 16 mutant to 84 wild type in the Zheng58/*pgl-sd* F_2_ population (*X*^2^_(16:84)_ = 0.55, *P* = 0.45) and Qi319/*pgl-sd* F_2_ populations (*X*^2^_(16:84)_ = 0.008, *P* = 0.92). We examined ears of Zheng58/*pgl-sd* F_1_ and that of Qi319/*pgl-sd* F_1_, but no obvious abortion of kernels were observed. In addition, no abnormality of F_1_ seeds germination or of F_2_ seedling establishment was found for these two crosses. Thus, we speculated that the mutation of *pgl-sd* could lead to some degree of abortion of gamete, which resulted in the observed segregation distortion.

### Initial mapping of *pgl-sd*

**Fig. 2:**
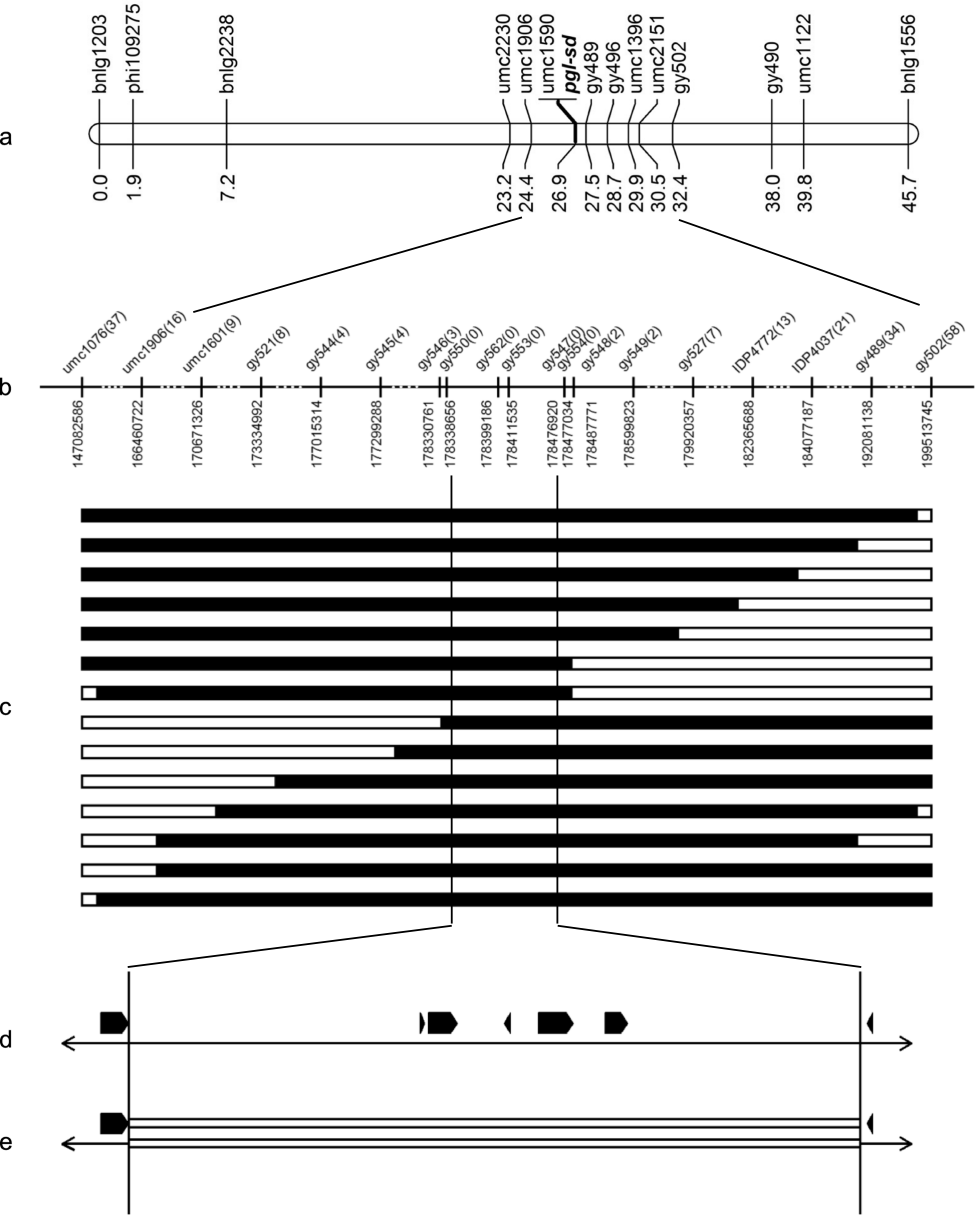
Mapping of *pgl-sd*. **a** linkage mapping of *pgl-sd* with Zheng58/*pgl-sd* F_2_ population (units = cM). **b** physical coordinates of markers on the chromosome 1 for fine mapping of *pgl-sd* with Qi319/*pgl-sd* F2 population based on RefGen_v5 (units = bp). The number of recombinant mutants between each marker and *pgl-sd* was showed in bracket following the marker. **c** genotypes of representative recombinants from the Qi319/*pgl-sd* F_2_ population. Black color indicates the *pgl-sd* genotype and white indicates heterozygous genotype. Codes of these plants from top to below are b3.08.01, b3.11.11, b3.67.09, b3.16.07, b3.31.07, b3.33.09, b2.11.09, b2.26.02, b1.67.10, b3.12.03, b1.13.09, b2.57.07, b3.01.18, and b1.66.10 respectively. **d** annotated genes in *pgl-sd* interval delimited by gy546 and gy548. **e** the deletion containing five genes in the* pgl-sd*

Initially, 5 mutant and 5 wild plants from the B73/*2*pgl-sd* population were genotyped with polymorphic markers equally distributed across maize chromosomes along with the parent lines. With these markers, *pgl-sd* was located into a region around bin 1.05 and 1.06 of chromosome 1. Because of low diversity observed between *pgl-sd* and B73, we then used the Population 1 to further construct a linkage map encompassing the *pgl-sd* mutation with polymorphic simple sequence repeat (SSR) markers located in the two bins or nearby. *pgl-sd* was finally mapped into a 5.5-cM interval delimited by the closest markers umc1906 and umc1396 (Fig. 2a). With molecular markers developed at the fine mapping stage, this interval was further resolved by gy496 and gy489 in the long arm direction (Fig. 2a).

### Fine mapping of *pgl-sd*

To fine mapping of *pgl-sd,* molecular makers umc2230 and umc1396 were used to identify recombinants between markers and *pgl-sd* (Fig. S1). Besides the 24 mutants from the Population1, other 591 mutants with no confused phenotype from the Zheng58/*pgl-sd* F_2_ were genotyped with the two markers. A total of 116 recombinants were obtained with one or both of the two markers loci carrying the allele(s) from the wild parent Zheng58. These recombinants were further genotyped with markers between umc2230 and umc1396 to reveal their detailed structure in this region. To increase resolution of the molecular map, we searched MaizeGDB database (https://www.maizegdb.org) and literatures for SSR or IDP markers possibly located in the target region. SSRs identified in the sequence of these region were also used to design molecular markers. These markers were screened for polymorphism between *pgl-sd* and B73, Zheng58, or Qi319. *pgl-sd* was further delimited to a ~ 6.4-Mb region enclosed by gy524 (at the position of 173.7 Mb on chromosome 1 of Zm-B73-REFERENCE-GRAMENE-4.0 (RefGen_v4)) and gy541 (180.0 Mb), with 7 recombinants between gy524 and *pgl-sd* and 2 between gy541 and *pgl-sd* being identified, respectively (Fig. S1). Recombination events were also identified by another SSR marker gy533 originated from the MISA search, which was located at 176.5 Mb on chromosome 1 of the RefGen_v4. But gy533 was not placed on the physical map because of the difficulty to distinguish the polymorphism.

Then, 387 mutants from Qi319/*pgl-sd* F_2_ were screened for recombinants with umc1076 and gy502 selected according to the genetic map constructed with Zheng58/*pgl-sd* F_2_. A total of 92 recombinants were obtained. *pgl-sd* was delimited to a region with 6.7-Mb physical distance delineated by the closest markers gy521 (171.7 Mb) and gy527 (177.8 Mb) originating from a literature (Jie et al. 2013) (Fig. 1). However, this interval could not be further resolved due to lack of more polymorphic markers.

To overcome the shortage of polymorphic markers, *pgl-sd* genome was sequenced on Illumina platform and the resulted reads were aligned to RefGen_v4 along with downloaded sequence reads of Zheng58 and Qi319 to identify variants. IDPs with length difference less than 4 bp between *pgl-sd* and Zheng58 or Qi319 in the interval delimited by gy524 (173.7 Mb) and gy533 (176.5 Mb) were screened for mapping *pgl-sd*. It was interesting that only 3 variants between Zheng58 and *pgl-sd* were obtained in this region, while 21 were gotten between Qi319 and *pgl-sd* under this threshold. We designed primers for 7 IDPs, 6 of which successfully detected the expected polymorphisms between Qi319 and *pgl-sd,* but none did between Zheng58 and *pgl-sd* as anticipated. With these markers, *pgl-sd* was further mapped to a 155.8-kb region bracketed by gy546 and gy548, with gy547 cosegregating with it (Fig. 1b)*.* 3 recombinants between gy546 and *pgl-sd* as well as 2 between gy548 and *pgl-sd* were observed, respectively. Coordinates for gy546, gy547 and gy548 were 176.20 Mb, 176.34 Mb, 176.35 Mb on chromosome 1 of the RefGen_v4. There were only 2 annotated genes in this region including *Zm00001d031078* and *Zm00001d031079*. However, careful inspection of this region revealed a large gap existing in the interval of the assembly of RefGen_v4, while no gap was present in the corresponding regions bracketed by gy546 and gy548 of both Zm-B73-REFERENCE-NAM-5.0 (RefGen_v5) and B73 RefGen_v3 (RefGen_v3). We aligned the sequence of target interval of RefGen_v5 with that of RefGen_v3 by using MUMer and found that these two assemblies agreed well with each other in this region (Fig. S2). According to the annotation of RefGen_v5, an unmapped contig B73V4_ctg98 from RefGen_v4 was found sharing high identity with the sequence of the target region of RefGen_v5. This was the reason why RefGen_v5 was used as reference to give physical positions of markers in the fine mapping of *pgl-sd* finally (Fig. 2 b).

There were 8 annotated genes in the interval of RefGen_v5, which included *Zm00001eb031850, Zm00001eb031860, Zm00001eb031870, Zm00001eb031880, Zm00001eb031890, Zm00001eb031900, Zm00001eb031910* and *Zm00001eb031920*. Among these 8 genes, *Zm00001eb031920* was in a tandem array with *Zm00001eb031910,* and both of them corresponded to the same annotated gene *Zm00001d031079* in RefGen_v4. Therefore, only *Zm00001eb031910* was considered in further studies for these two gene models. Marker gy547 was located at the interval between *Zm00001eb31900* and *Zm00001eb31910*. Then, we reanalyzed the sequence reads of *pgl-sd*, Zheng58 and Qi319 using RefGen_v5 as the reference genome in order to find more variants for developing markers to saturate the target region. Intriguingly, genotype information was missing for *pgl-sd* at many variant positions in this region reported by GATK [35], which was confirmed by the observation that there were no mapped reads for *pgl-sd* in many parts of the region. Three new markers were obtained, including gy550, gy553, gy554. gy550 was designed from *Zm00001eb031850*, the gene nearest to gy546 in the target interval and gy553 was from the sequence between *Zm00001eb31880* and *Zm00001eb31881*. gy554 was developed from the sequence between *Zm00001eb31900* and *Zm00001eb31910* like gy547, but it was a little closer to gy548 than gy547. However, all of the three markers cosegregated with *pgl-sd* (Fig. 2), with gy553 as a dominant marker having no specific amplification in mutant plants. These results indicated that it was likely to be difficult to further resolve the region around *pgl-sd* mutation though recombinants could be found between gy546 and gy548.

### A large deletion in pgl-sd leading to the phenotype of pgl-sd mutation

Among the 7 annotated genes in the target region, *Zm00001eb031870/Zm00001d000230* had *At4G18370* as the ortholog in *Arabidopsis* (http://www.maizeGDB.org) which encodes Degradation of periplasmic proteins 5 (Deg5), a protein located in chloroplast thylakoid lumen [44]. It was the only gene which had its high expression present in only leaves according to the RNA-seq expression data in maizeGDB (https://www.maizegdb.org) (Fig. S3). Marker gy562, developed from *Zm00001eb031870*, cosegregated with *pgl-sd* (Fig. 2b). So we speculated that *Zm00001eb031870* was the candidate gene responsible for *pgl-sd*. To test the hypothesis, reverse transcription PCRs (RT-PCRs) / reverse transcription quantitative PCRs (RT-qPCRs) were conducted to examine the expression of these 7 genes in leaves of *pgl-sd* and the wild line Qi319 (Table 1). All primer pairs designed from *Zm00001eb031910/Zm00001d031079* failed to specifically amplify products of expected size. The expression of *Zm00001eb031850/Zm00001d031078* was too low to be detected in leaf, but it was high in the root of B73 (data not shown), which was consistent with RNA-Seq expression data in maizeGDB (https://www.maizegdb.org). As expected, the transcription of *Zm00001eb031870* was significantly down-regulated in *pgl-sd* relative to Qi319. However, the expression of *Zm00001eb031880/Zm00001d000229* was detected in all lines (B73, Zheng58 and Qi319) except *pgl-sd,* and the expression levels of all the other 3 genes were also significantly lower in *pgl-sd* than that in Qi319 (Table 1).

**Table 1:** RT-qPCR analysis of candidate genes for *pgl-sd*

gene	primer	Relative expression^a^		P^b^
		YLP	Q319	
Zm00001eb031850	gy555	u	u	-
Zm00001eb031860	gy556	0.25 ± 0.05	0.36 ± 0.05	0.05
Zm00001eb031870	gy557	u	1.56 ± 0.16	-
Zm00001eb031880^c^	gy565	-	-	-
Zm00001eb031890	gy559	2.33 ± 0.77	7.14 ± 1.57	0.02
Zm00001eb031900	gy560	low	0.28 ± 0.05	-
Zm00001eb031910^c^		-	-	-

^a^average relative expression ± sd with FPGS used as reference gene, “u” and “low” indicated "undetermined" and CT > 34 in q-PCR analysis, respectively. ^b^P value for two tail T-test of expression difference between *pgl-sd* and Qi319. ^**c**^Expression differences of *Zm00001eb031880* and *Zm00001eb031910* were not investigated due to amplification failure of the former in leaf of *pgl-sd* and no appropriate primer pair were obtained for the latter

**Fig. 3:**
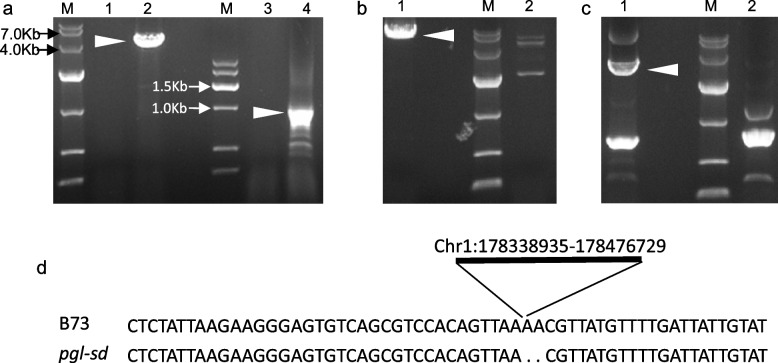
The structure variation in *pgl-sd*. White triangles indicate expected PCR products. **a** amplification of Zm00001eb031870 with the primer pair gy573. **b**, **c**, validation of the deletion in* pgl-sd* with gy598 and gy599, respectively. **d** sequence alignment of gy598 amplificon from *pgl-sd* with the target region of RefGen_V5. M, molecular weight marker. For 1 and 2, genomic DNA of *pgl-sd* and Q319 were used, respectively. For 3 and 4, cDNA of *pgl-sd* and Q319 were used, respectively

We then tried to amplify the full-length genome sequence and the coding sequence (CDS) of *Zm00001eb031870* in *pgl-sd* and Qi319 with the primer pair gy573. However, PCR products of expected size were only obtained from Qi319 (Fig. 3a). Impressively, same results were gotten with three additional primer pairs designed from different parts of this gene (data not shown), indicating that *Zm00001eb031870* was possibly deleted in *pgl-sd*. Combining these results with the gene expression analysis, gy553 being a dominant marker, and many missing data for *pgl-sd* at variant positions in the target interval together, we suspected that a large deletion might exist in the target genome region of the mutant. Then, we scrutinized regions beyond *Zm00001eb031870* in the whole interval by developing additional primer pairs, including all the other 6 genes and intergenic regions following *Zm00001eb031850* and preceding *Zm00001eb031910.* All these primer pairs amplified products with expected size from Qi319 but did not from *pgl-sd*, except gy580 derived from the region between *Zm00001eb031850* and *Zm00001eb031860* and gy586 from *Zm00001eb031890*, which generally agreed with our hypothesis.

To further validate this hypothesis and determine the precise position of the possible deletion, Manta was used to detect signals of SVs in the targeted region with the sequence read mapping result of *pgl-sd*. However, no meaningful information was attained. Then we tried Breakdance. Fortunately, a 137,794-bp deletion starting from 178,338,866 bp and ending at 178,476,727 bp on the chromosome 1 of RefGen_v5 was identified with 4 supported reads. To catch more supported information, we assembled sequence reads of *pgl-sd* by using SOAPdenovo and found a 8204-bp contig C52375234 (data s1) matching the deletion-supporting reads from the SV analysis using Breakdancer. Blastn search with C52375234 against RefGen_v5 demonstrated that its right part (from 1 to 6,010 bp of its reverse complement) matches the region from 178,332,925 to 178,338,934 of B73 chromosome 1 completely and its left part (from 6,009 to 8,024 bp of its reverse complement) aligned with the region from 178,476,727 to 178,478,922 of B73 chromosome 1 with 100% identity, repectively, suggesting a deletion starting from 178,338,935 bp and ending at 178,476,729 bp on the chromosome 1 in *pgl-sd* compared with B73, which was consistent with the prediction from Breakdancer but with a little difference at the exact starting position. The primer pair gy598, flanking the predicted deletion, did amplify products of expected size from genome DNA of *pgl-sd* but did not from Qi319 due to too larger size of the segment flanked by gy598 (Fig. 3b). Similar results were brought for the primer pair gy599 which was internal to gy598 though it demonstrated worse specificity than gy598 (Fig. 3c). Sequencing amplicons from *pgl-sd* with gy598 resulted an anticipated 6,119-bp sequence, which matched the C52375234 with 100% identity. Thus, these data confirmed our hypothesis (Fig. 3d, Data S1).

### Mechanisms for the phenotype of *pgl-sd*

**Fig. 4:**
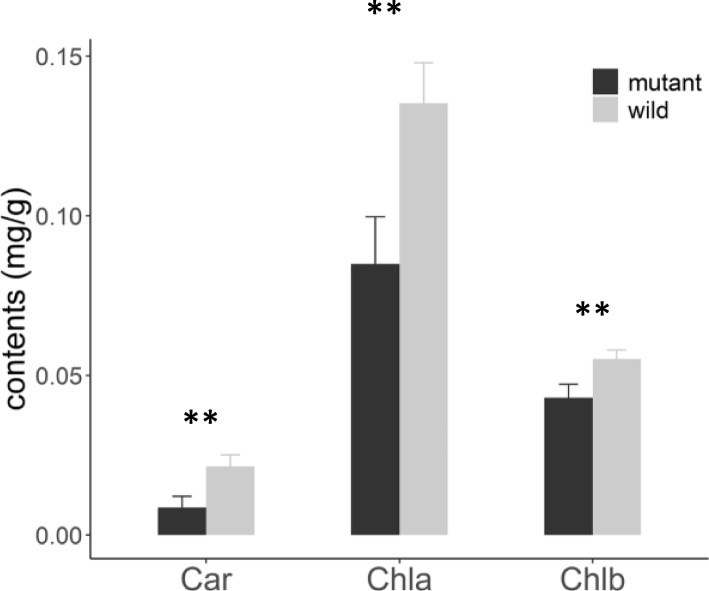
Differences of leaf photosynthetic pigment contents between mutants and wild plants. Plants were sampled from B73/*2*pgl-sd* BC_1_ population. Data was showed as mean ± sd (*n* = 8 for mutants and 9 for the wild-type, respectively). ** indicates *P* < 0.01

Because *pgl-sd* mutants exhibited pale green leaves, we examined whether contents of chlorophyll a (Chla), chlorophyll b (Chlb) and carotenoids (Car) were changed in *pgl-sd*. It was detected that contents of all of three pigments were significantly reduced in the mutant compared with the wild-type at early seedling stages (*P* < 0.01) of the B73/2*pgl-sd population (Fig. 4). The content of Chla, Chlb and Car of the mutant was 62.8%, 77.9% and 40.2% compared to the wild-type, respectively. Thus, the mutation of *pgl-sd* reduced the accumulation of these major classes of photosynthetic pigments.

**Fig. 5:**
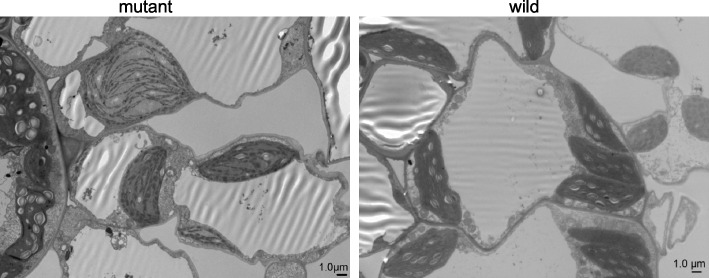
The structure change of chloroplasts in the mutant

Further, scrutinization of seedling leaves with electron microscopy revealed that chloroplasts in mesophyll cells of the mutant were larger in size than that of the wild-type (Fig. 5). Chloroplasts of mesophyll cells in the mutant also displayed irregular shapes and had small granal stacks compared with that in the wild-type. These results suggested that the *pgl-sd* mutation likely affected the development of chloroplast directly, which then resulted in the reduction of photosynthetic pigments accumulation.

RNA-Seq analysis was also conducted to divulge effects of the mutation of *pgl-sd* on gene expression in the developing leaves. In the differentially expressed gene (DEG) analysis, a total of 345 genes were identified under the threshold mentioned in the methods. No over-represented Gene Ontology (GO) items were observed with adjusted P-value < 0.05 in GO enrichment analysis of DEGs, but 82 items were enriched under threshold of *P*-value < 0.05. The top three significantly enriched items classified as Biological Process (BP) were GO:0009405 (pathogenesis), GO:0009750 (response to fructose) and GO:0051341 (regulation of oxidoreductase activity), respectively. The top three items classified as Cell Component (CC) included GO:0032040 (small-subunit processome), GO:0015629 (actin cytoskeleton), GO:0000275 (mitochondrial proton-transporting ATP synthase complex-catalytic core F(1)). The top three items of Molcular Function (MF) comprised GO:0016831 (carboxy-lyase activity), GO:0004612 (phosphoenolpyruvate carboxykinase (ATP) activity) and GO:0008483 (transaminase activity). No information clearly related to the trait pale green leaf was obtained from these data.

Among genes located into the deletion region, *Zm00001eb031870*/*Zm00001d000230, Zm00001eb031880/Zm00001d000229* and *Zm00001eb031900/Zm00001d000227* were expressed in the wild-type but no expression was detected for them in the mutant as expected (Table 2). However, expression of *Zm00001eb031860/Zm00001d000231* was detected in the mutant at a low level in comparison with the wild-type. Similarly, the expression of *Zm00001eb031890/Zm00001d000227* was also observed in the mutant though it was significantly lower than that of the wild-type (Table 2). These data coincided with the results of RT-qPCR. It was possible that the expression of highly homologous genes interfered with the exact detection of *Zm00001eb031860* and *Zm00001eb031890*. In fact, *Zm00001eb031890* shared high identity to *Zm00001eb159540* and the cDNA of *Zm00001eb159540* was amplified with a primer pairs designed from *Zm00001eb031890* (data not shown).

**Table 2:** RNA-seq analysis of the expression of genes located in the *pgl-sd* deletion

Gene ID(RefGen_v4)	Gene ID(RefGen_v5)	Expression(FPKM)^a^		P^b^
		Mutant	Wild	
Zm00001d000231	Zm00001eb031860	0.04 ± 0.01	0.07 ± 0.02	0.71
Zm00001d000230	Zm00001eb031870	0.00 ± 0.00	19.78 ± 4.36	0.00
Zm00001d000229	Zm00001eb031880	0.00 ± 0.00	4.09 ± 0.97	0.00
Zm00001d000228	Zm00001eb031890	1.36 ± 0.35	6.68 ± 3.27	0.03
Zm00001d000227	Zm00001eb031900	0.00 ± 0.00	37.63 ± 5.03	0.00

^a^data were presented with mean ± se. ^b^*P*-value from analysis of DESeq2

*Zm00001eb031860/Zm00001d000231* could be assigned to GO:0003678, classified as a MF item with the description of DNA helicase. Search against the InterPro protein signature databases (https://www.ebi.ac.uk/interpro) showed that Zm00001eb031860 had a Pif1-like helicase domain. It was found that both GO:0031570 and GO:0044774 in the BP, with the description of “DNA integrity check point”, were enriched with P-value = 0.07, indicating that deletion of *Zm00001eb031860* might affect the DNA repair system.

*Zm00001eb031870/Zm00001d000230* could be assigned to GO:0009535, a CC GO item with the description of “the pigmented membrane of a chloroplast thylakoid”. *Zm00001d008209*, a gene assigned to GO:0009535, was significantly up-regulated at the transcriptional level in the mutant compared with the wild-type. According to the annotation of maizeGDB, *Zm00001eb031870* might be involved in photosystem II (PSII) repair. The GO item GO:0009654 in CC, with description “photosystem II oxygen evolving complex”, was enriched with P-value = 0.07. 2 DEGs were gouped to this GO item, among which the expression of *Zm00001d049390* was not detected in the mutant. In addition, 2 genes assigned to GO:0009765 with the description of “photosynthesis, light harvesting” was differentially expressed in mutants compared with the wild-type. It was of note that both *Zm00001eb031890/Zm00001d000228* and *Zm00001eb031900/Zm00001d000227* might participate in chloroplast development too. Search against InterPro database revealed that *Zm00001eb031890/Zm00001d000228* had sequence similarity to the pthr10566 which was described as “Protein Activity of BC1 Complex Kinase 8, Choloplastic”. *Zm00001eb031890* was grouped to GO:0009941 (“chloroplast envelope”) and GO:0046467 (“membrane lipid biosynthetic process”). *Zm00001eb031900/Zm00001d000227* could also be assigned to GO:0009941 and it was homologous to *AT1G30120* which encodes a part of plastid pyruvate dehydrogenase complex.

Search with Conserved Domain Architecture Retrieval Tool (CDART) (https://www.ncbi.nlm.nih.gov/) demonstrated that *Zm00001eb031880/Zm00001d000229* had sequence similarity to a cotton fiber-like protein (DUF761). In *Arabidopsis,* DUF761-containing proteins likely had a role in plant development and disease resistance [56]. It was interesting that GO:0009405, a BP item with description of “pathogenesis”, was the most significantly over-represented item (P-value = 0.0008). In addition, items GO:0010112 (“regulation of systemic acquired resistance”) and GO:0009870 (“resistance gene-dependent”) in the BP were also significantly enriched.

Taken together, the deletion in *pgl-sd* affected its chloroplast development, which might lead to the decrease of photosynthetic pigment contents in leaves. But not many DEGs and no high enriched GO items were identified in our research. The possible reason might be that the RNA samples were prepared from plants of a B73/2*pgl-sd population. The genetic background differences between individuals resulted in high variations of gene expressions within the mutant or wild pools, which reduced the power for the DEG detection. But the deletion including three genes possible related to the structure and function of chloroplast affected the expression of several genes involved in the structure and function of chloroplast. Besides these, the mutation might also affect the DNA repair system and the disease resistance as well.

## Discussion

To date, 8 green-leaf-color genes have been isolated in maize, which included *Elm1*, *Elm2*, *Chr.1-ClpP5*, *Oy1*, *Oy2, Vyl*, *Ygl-1*, *Zb7* [14, 32, 41, 42, 52, 54], none of which was located in the deletion reported here, indicating that *pgl-sd* involved in leaf color-related genes different from those reported. There were 5 annotated genes located in this deletion according to the annotation of B73 genome (https://www.maizegdb.org), 3 of which might be related to structure and function of chloroplast, including *Zm00001eb031870/Zm00001d000230, Zm00001eb031890/Zm00001d000228* and *Zm00001eb031900/Zm00001d000227*. In *Arabidopsis* protein database (https://www.arabidopsis.org/), Zm00001eb031870 shared highest identity with At4g18370, known as Deg5. In *Arabidopsis*, 4 Degs are located in chloroplast, three of which, Deg1, Deg5 and Deg8, are present in thylakoid lumen [44]. *Aarabidosis* mutant *deg1* was small, and had thin and pale green leaves compared with the wild-type [4, 20], a phenotype sharing by *pgl-sd*. But *deg1* flowered earlier than the wild-type whereas *pgl-sd* flowered later*.* But loss of function of *Deg5,* the ortholog of *Zm00001eb031870,* in *Aarabidosis*, has no visible effects on normal conditions [4, 44]. *tcm5*, a rice *Deg* mutant, also exhibited albino phenotype and defective chloroplasts [57]. *Zm00001eb031900/Zm00001d000227* was orthologous to *AT1G30120* which putatively encoded a plastid pyruvate dehydrogenase complex E1 component subunit beta (ptPDC-E1-β). The ptPDC provides acetyl-CoA and NADH for fatty acid biosynthesis in plastids [5, 21].The *floury endosperm19* (*flo19*), a *ptPDC-E1-α1* mutant in rice, showed low plant height and slow growth throughout the entire growth period rative to the wild-type, a phenotype reminiscent of *pgl-sd*, in addition to opaque of the interior endosperm [25]. It was surprising to observed that *Zm00001eb031870* and *Zm00001eb031900* were the only two core genes revealed by pan gene analysis of the deletion region with data of MaizeGDB (https://www.maizegdb.org) (data not showed). These data suggested that *Zm00001eb031870* and *Zm00001eb031900* might be the major causative genes for the observed phenotype of *pgl-sd*. However whether the deletion of *Zm00001eb031890* contributes to specificity of the phenotype of *pgl-sd* remains to be elucidated.

Besides the aforementioned three genes, the *pgl-sd* deletion region contained *Zm00001eb031860* and* Zm00001eb031880,* which were possibly related to DNA repair system and plant disease resistance, respectively. Though limited information was obtained from our RNA-seq data, it was observed that transcription of some genes in related pathways were affected in the mutant. But the traits we examined only included plant height, leaf color, and plant growth. Thus, it is necessary to find a appropriate set of traits to confirm the possible effects of the deletion of these two genes.

SVs are crucial for genome evolution and abundant in genomes, especially for species like maize, which, as an ancient tetraploid, experienced many times of duplication events, but few validated SVs have been reported in maize. The finding of the deletion of *pgl-sd* provided valuable information for functions and mechanisms of SVs in maize genome. Several mechanisms have been proposed for occurrence of SVs, including non-allelic homologous recombination (NAHR), microhomologous recombination (MHR), non-homologous end joining (NHEJ), microhomology-mediated end join (MHMEJ) and microhomology-mediated break-induced replication (MMBIR), etc.[6, 17]. NAHR requires long stretches of homologous sequences flanking the genomic region of SVs. MHMEJ or MMBIR is characterized by short homologous sequences (< 70 bp) at the break-joint position, whereas MMBIR was prone to result in complex SVs [6, 17]. For *pgl-sd,* no long homologous segments were observed flanking the deletion, but a 4-bp same nucleotide, A, were present at the break-joining point (Fig. 3), indicating that MHMEJ might lead to the deletion.

## Conclusions

In this study, we identified a 137.8-kb deletion through map-based cloning of *pgl-sd*, in which no maize leaf color associated genes were reported before. This deletion led to abnormality of chloroplast development, reduced contents of photosynthetic pigments in leaves, and affected the expression of genes involved in the structure and function of chloroplast. Three genes in this deletion were possibly related to the plastid development with roles different from that of other isolated maize leaf color associated genes.

As *Zm00001eb031870,* an ortholog of *Arabidopsis Deg5*, and *Zm00001eb031900,* putatively coding ptPDC-E1-β, were the only core genes in the identified deletion, the mutation effects on maize phenotype suggested these two genes may be necessary for normal maize development*.* The reports on the function of Degs and ptPDCs in other plants also point out the value of exploring the use of both genes in breeding maize varieties with higher yield potential and better stress tolerance to extreme environments, especially characterized by high temperature and light, in the future.

## Methods

### Plant materials and phenotyping

The spontaneous mutant *pgl-sd* was isolated from a breeding population. In consideration of possible effects of genetic backgrounds on phenotypic expression of the mutation of *pgl-sd*, the mutant plants were crossed with three elite wild inbred lines, including B73, Zheng58 and Qi319. B73 and Zheng58 belong to the Stiff Stalk group, and Qi319 is a line derived from mixed origin. The BC_1_ population, B73/*2*pgl-sd*, was developed by backcrossing B73/*pgl-sd* F_1_ individuals with *pgl-sd*. F_2_ populations were created for the other two crosses.

Phenotypes of the mutant *pgl-sd*, the three wild inbred lines and their F_1_ progeny were investigated in green house at seedling stages (from emergence stage, V0, to vegetative phase 3 stage, V3), and in the field at all growth stages. For segregation analysis, plants of BC_1_, F_2_ populations and their parents were grown in plastic trays in a green house and leaf color was evaluated at stages from V0 to V3.

### Molecular markers development and genotyping

SSR and IDP markers were retrieved from maizeGDB database (https://www.maizegdb.org) for mapping of *pgl-sd*. At the fine mapping stage, IDP and SSR markers listed in the two papers [19, 29] were used, but primer pairs were redesigned in most cases. The nucleotide sequence around the target region of RefGen_v4 were also used to search for potential SSR with MISA [2] for fine mapping of *pgl-sd*.

At the final stage of the mapping study, *pgl-sd* was resequenced to develop more IDP markers. Leaf samples of *pgl-sd* seedlings were used for genomic DNA extraction. A 350-bp insertion size library was constructed and sequenced with Illumina NovaSeq6000 at Novogene (Tianjin, China), yielding a total of 276,713,444 150-bp paired-end reads (41.12G base). Sequence data of Zheng58 (PRJEB30082) and Qi319 (PRJNA609577) were download from EBI (https://www.ebi.ac.uk). Reads with poor quality was filtered with Trimmomatic [3] and evaluated with FastQC (https://www.bioinformatics.babraham.ac.uk/projects/fastqc/). Clean reads were mapped to the RefGen_v4 or RefGen_v5 by using BWA mem [26] with default parameters. The mapped reads were sorted with SAMtools (Li et al. 2009) and marked duplication with picard (https://broadinstitute.github.io/picard/), then subjected to GATK (HaplotypeCallerfunction) [35]) for variant calling. Raw indels were filtered with expression "QD < 2.0 || FS > 200.0 || ReadPosRankSum < -20.0". Primers were designed using Primer3 [38] and synthesized at Sangon (Qingdao, China). Primer information for markers used for genotyping and other analyses in this study was listed in (Table 3).

**Table 3:** Primers used in the study

NAME	Forward primer	Reverse primer	Anealing Temp.(^o^C)	Type	F Start Pos.^a^	R start Pos.^b^	Gene
gy489	ACATCTTTCGTTCGTAGACCGT	CCGACTCTTCTGACCCAGTCC	55	IDP	192,081,138	192,081,405	
gy490	ACTCCGCTTCATCGACTCAT	TTGGCACTCAATCACCAAAA	55	IDP	"204,958,969	204,959,142	
gy496	AAGCTCGACTCTTTTGGTTC	CTCTTTTCATTTACCCCTGCTAC	55	SSR	193,783,975	193,784,132	
gy502	CTGCACAAGAACGTGAGTGA	AGTCATCAAAAGATCTACATGC	55	SSR	199,513,745	199,513,899	
gy521	ATCTTAATCTTGTGCGGGTTCA	CAAAATTTTAACAACACCTCCCCT	55	SSR	173,334,992	173,335,251	
gy524	AAAATACCAGCATGAGGGAT	GTGAACAAATTAACTAATGAAAC	55	SSR	175,789,629	175,789,904	
gy527	ATCATGACAAAAGGCAAGTGAC	GGCCCAAAACACAGTAGAACC	55	SSR	179,920,357	179,920,590	
gy541	GACATTTAAGGTGCCCACGA	CTCAAGAAAGGTTAAACGGG	55	SSR	182,124,940	182,125,239	
gy544	AGCAATTAGTTTACAAATTTCCA	CCTTTCTGTTTACTTCGCCTG	55	IDP	177,015,314	177,015,562	
gy545	TGGAGAAGTTAGCCACAACC	TACATGTTTGTGGCTTGAACT	55	IDP	177,299,288	177,299,483	
gy546	CTAGCAAAATTGTAGATGCAC	AAGAGACAATCTATGGCTT	55	IDP	178,330,761	178,331,000	
gy547	TCATGGACTATGGTGTGACGA	TCGATCGGACAATTATGGAC	55	IDP	178,476,920	178,477,058	
gy548	TTGTGCTCCATATCCTGTCC	TTTCGTCAGCGATCTACCTC	55	IDP	178,487,771	178,487,962	
gy549	CCAATAGCTTGTAATGGTT	TATCGTCTCTACAAGTCCT	55	IDP	178,599,823	178,599,969	
gy550	GATGCAAAATTTCGCCGAT	ATATAGCTAGCCAACAAAGGG	55	IDP	178,338,656	178,338,868	
gy553	ACTCGCAAAGATTTCCTG	CCATAGATCCCTAGCTCC	55	IDP	178,411,535	178,411,726	
gy554	ACATAGTCCATAATTGTCCGAT	TAGCGTTAAACCAACTACCAG	55	IDP	178,477,034	178,477,154	
gy562	TTGGAGTATAAATTAAAAGACTA	ATCGAAAAGAGAATTGATT	55	IDP	178,399,186	178,399,334	
gy555	CGAGATCATCGGAGGAATCCTG	GCCAGCGTGTCCAGCACCGTC	60				Zm00001eb031850
gy556	GAGTTGCCACACACTAACCAA	TCACAAACCCAACTAACAGCA	60				Zm00001eb031860
gy557	CGATCCGTGGGGCTATACAGACA	ATCCCAGATCCTTTACGTGTGA	60				Zm00001eb031870
gy559	AATAGCTTTCAAGAGTGCCTA	ATCCTCTCCGATTGTAGCAAG	60				Zm00001eb031890
gy560	ATCCAAGTCAGGCGGCCATGA	ACATCTTCACCCATGACGCACAC	60				Zm00001eb031900
gy565	CCTCCTCCAGCTGTAGCCTCA	GCTGTCGGACGAGGAGCTGAAC	60				Zm00001eb031880
gy573	TGACCTTCGCTTCTATACTGTCT	TCAGATGTCCAGCACCGTCGAT	66		178,395,427	178,399,988	
gy580	TGACCTTCGCTTCTATACTGTCT	TCAGATGTCCAGCACCGTCGAT	57		178,393,468	178,393,571	
gy586	TGACCTTCGCTTCTATACTGTCT	TCAGATGTCCAGCACCGTCGAT	57		178,417,639	178,417,793	
gy598	TCCCCGTTCCAATCCATGTTCCCACCC	TGCCATGCACGCCGCAAACTATACCG	68		178,334,160	178,478,070	
gy599	TGACCTTCGCTTCTATACTGTCT	TCAGATGTCCAGCACCGTCGAT	60		178,335,787	178,477,075	

^a^start position of forward primer on the chromosome 1 based on RefGen_v5; ^b^start position of reverse primer

PCR amplifications were performed using TransGen (Beijing, China) *EasyTaq* DNA Polymerase for page with annealing temperature being set to 55 centigrade. PCR products were separated with electrophoresis on 8% (W/V) polyacrylamide gel with a acrylamide to bisacrylamide ratio of 19:1 (W/W) or 39:1.

### Linkage map construction

A linkage map was constructed with MAPMAKER 3.0b [24] in the initial mapping. Linkage groups were determined with a minimum logarithm of odds (LOD) score of 3.0 and max distance 50 cM. Recombination frequency was computed with Haldane’s mapping function. Linkage maps were draw with MapChart [47].

### Detection and validation of SVs

Mapping data of genome sequencing reads for *pgl-sd*, Zheng58 and Qi319 based on RefGen_v5 were used to detect SVs in *pgl-sd* compared with B73 by using both Manta [9] and Breakdancer [12], respectively. The genome assembly of *pgl-sd* was performed by using SOAPdenovo (SOAPdenovo-63, with K being set to 63) [33] with cleaned reads subjected to duplication removing. The final assembly was utilized to confirm possible structure variants predicted by the software aforementioned when necessary. PCRs for experimental SV validation were conducted with Vazyme (Nanjing, China) Phanta Flash Master Mix following the factory’s manual and PCR products were separated with 1% (W/V) agarose gel.

### RNA extraction, reverse transcription and RT-qPCR analysis

The first leaves of *pgl-sd,* B73, Zheng58, Qi319 plants at V2 stages were harvested for total RNA extraction by using a Aidlab's plant RNA extraction kit RN09 (Aidlab, Beijing, China). RNA was reverse transcribed to cDNA using Accurate Biology's RT Kit (AG accurate Biology, Changsha, China). RT-qPCR was performed with a SYBR Green Real-Time PCR Master Mix (Accurate Biology, Changsha, China) using The Applied Biosystems 7500 Real-Time PCR System (ThermFisher Scientific) following the comparative CT experiment protocol of the manufacturer. Experiments were performed with three independent RNA samples and three technical replicates and *folypolyglutamate synthase* (*FPGS*) was used as the reference gene [34]. Relative gene expression was calculated as described by Livak and Schmittgen [30].

### Measurement of chlorophyll and carotenoid contents

Because the population from which *pgl-sd* originated was not available, plants from the B73/*2*pgl-sd* population were used in the leaf pigment measurement as well as microscopy analysis and RNA-seq. Leaves of plants at V2-V4 stages (approximately 200 mg fresh weight for each plant) were cut into pieces and then submerged in a 10-ml solution of 2:1 (V/V) 95% acetone to ethanol for 48 h at 26 °C under dark conditions. OD values of these extracts were measured with Nanodrop2000 (ThermFisher Scientific) at 663, 645, and 470 nm, respectively. Contents of Chla, Chlb, and Car were estimated as described by Guan et al.[14].

### Transmission electron microscopy

Plants at V2 to V3 stages, which grew in a greenhouse, were used for transmission electron microscopy analysis. Sample sections were prepared according to the description of Guan et al. [14] and observed using a Hitachi transmission electron microscope H-7500.

### RNA-Seq Analysis

Leaves of plants at growth stages from V2 to V4 were harvested for RNA-Seq analysis. Six mutants or six wild plants were pooled together as one biological sample for one phenotype group and three independent samples were prepared for each phenotype group. Sequencing was performed at Novogene (Tianjin, China). Clean reads were mapped to RefGen_v4 using Hisat [22]. DEGs between the mutant and the wild group were determined with adjusted P-value < 0.2 and fold change > 2 using the R package DESeq2 [31]. GO enrichment analysis of DEGs was implemented by using the R package clusterProfiler [50] with maize-GAMER used as the GO annotation [49].

### Statistical and graph drawing soft

All data in the work were analyzed with R (https://cran.r-project.org/) and visualized with R package ggplot2 [48] except otherwise mentioned.

## Supplementary Information

**Additional file 1.**

**Additional file 2.**

**Additional file 3.**

## Data Availability

All data generated or analyzed during this study are included in this published article and its supplementary information files. The sequencing data are available at NCBI SRA database under the project of PRJNA823837.

## Abbreviations

BP: Biological Process
Car: Carotenoids
CC: Cell Component
CDS: Coding sequence
Chla: Chlorophyll a
Chlb: Chlorophyll b
Deg: Degradation of periplasmic protein
DEG: Differentially Expressed Gene
FPKM: Expected number of fragments per kb of transcript sequences per Mb sequenced
GO: Gene Ontology
IDP: Indel polymorphism
MF: Molcular Function
pgl-sd: Pale green leaf-shandong
ptPDC: Plastid pyruvate dehydrogenase complex
ptPDC-E1-β: Plastid pyruvate dehydrogenase complex E1 component subunit beta
QTL: Quantitative Trait Loci
RefGen_v3: B73 RefGen_v3
RefGen_v4: Zm-B73-REFERENCE-GRAMENE-4.0
RefGen_v5: Zm-B73-REFERENCE-NAM-5.0
RT-PCR: Reverse transcription PCR
RT-qPCR: Reverse transcription quantitative PCR
SNP: Single nucleotide polymorphism
SV: Large scale structure variant
