# Supplementary material for: A large deletion conferring pale green leaves of maize
Source: BMC Plant Biol. 2023 Jul 14;23:360. doi: 10.1186/s12870-023-04360-2 (PMC10347855; doi:10.1186/s12870-023-04360-2)
Supplement: Supplementary file 1 — Additional file 1. [file 12870_2023_4360_MOESM1_ESM.doc]

>C52375234

GTACGACGGCGGCGGCGGCGGCGGCTACGTCCTCGTCGACCTGTCCCTGACGCTGGGCCC

GACCGCGACGCCACACCCTGCCTCCTCCTCCTCTCCTGAGCCTGACCACACACCAGCCGC

CGCCGCCGCCGGTTCCAGTTCCGCCGGCGTGCGGCTGTTCCCGTGCCTCTTCTGCAACAA

GAAGTTCCTCAAGTCGCAGGCGCTCGGCGGCCACCAGAACGCGCACAAGAAGGAGAGGAG

CGTGGGGTGGAACGCCCACCTCTACCTCCCGCCGCCGGCCGCGGACACGGCGTCGGCGTC

GGTCGCCGGCGGCGCCGCCGCTCGTGCCGCGACGACGCCCGTCGGCAGCAGCCACGCGCT

GCCGATGATCCAGGCCGTCTCGCACTCGTGCCACCACCATGAGCACCACCACCGGGACGG

CGGCGCGGCGGCGCCGTTGTTGGAGACGGCGTGGTGGTCGTCGTGCGCGGAGGAGAAACA

GCAGAGGCACCCCGTTGACCTCAATCTCAGGCTCTAGCTGCTAGCGCTGTCATATCCCCT

GCTTGTGTGCGTAATATATAAATCCTGAAGGAGTACGTAACGTACCGTACGTGCTGTCGA

GGTAGGGTGAGGCGTCTCGGTGTGCCTGTGCAAAAGCGTGCGAGGCCCATGGGCCATGGC

CCAGCATTCTATGATTATGTGTCGTCGCAACGAGCACGCACACTTTATAGTTTTATGGTG

CCAATTATATGCCTCTTTATTTCATTCTGAGCGAGAGGTGTAAGAAAGCACCCGCTGGCC

GGTGCTGTGGACTGGAGAATGTTTTATTTGCATGCACGCACGCCGCAAACTATACCCAAG

TCTTTGTTTATTTGCCATGCACGCCGCAAACTATACCGAATAATGTTTTATTTGTATGCA

TGATATCCGCCGCATCATTGAGTAGCCTCACACACACATACGATGTAAGATATCCGCCGC

ATGTCGAAGGCTTTGGCGGAGAACGTTGGGTGACAAGAGAATTAGCACTATCGACCTTGC

ATTCTTCACCGAGTGTCTCAAACACTCGGCAAATAACGCTCGACAGATATTTTATCGACA

AATAGTTCTTTACCGAGTATTTTTTGGGGGCACTCAGCAAATACTTTGTCGAGTGTCAAA

AAACACTCGGCAAATTAACATCGAAAAAAAAACATCAAAACAATTTTCTAAATTAGGGAT

TAGGAGAACAACACCCTAACCATCACCATTGCCCTACCCATCGTCCTATCATTTTTTCAC

CATTTTTTTAACTGAATTCAGGTGTTTTGTGACTGGTAAGATTTGAACTCGCAACCTCGG

CGAAAAAACCCTATTTATCGTCCTATCATTTGAATTAATTTACTGCTTTAAAATACGATT

TTTAAATTATCTTTGCGTAGTGTTGAAAAAAAACACTCGGCGGCAAAGAAACTCTTTGTC

GAGTGTCAAAAATAAATAACTCGGTAAAGAGCCGGATTCCGGTAGCGCATGGTAGAGAAA

ATTCCTTGCTATTTAAAATTAAACAAATGATTTTTTTTCCATGGATCCGCTCCAATTCAT

TGCAACCAAACCACACCCCCACAGGCAACATTCATTTACAAGTTTATGGAAGAAGGAAGA

TGCTTCTACGTGCTAGACTAACATTTCAAGTAGAGATCGTAGAAAATAGACAGTGCCTGT

GTGTTAGTGTGGACGCTTGCTTAGTTCACGGATGCCAGCAATAATTGGGGATTTGATCTC

CCTTTCCCTTATGCGGTTTCTTGTTGGTTAGCGTTAAACCAACTACCAGAGAGAGAGAGA

GGGGGGAGAGAGAGAGAGCATCAAGTGTAGAGCATTATTTGCCCTTGTCAGATGTCCAGC

ACCGTCGATCGGACAATTATGGACTATGTGGTACATTTCCACTTCCATCACAAAGATCTA

AAACTTGATATGTGGGGCCTAATCCTGCTGGTGCATGCACTCATCATCCGCTGAGAGATT

GGTCGTCACACCATAGTCCATGACATCCAGTGACGGATCTAGGATTGATCCAAGAGGGGT

TACTAAGCTAAGGCTATAAAATATTTTAAACCAAACAAAAAATATAATTGATGTTTAATA

TGACACAAGAAGATATAATTTAAACATTCAAAAAATATGTAGTCCAAAAATAACTCGATA

CGAATTCTAGAAATACAATAATCAAAACATAACGTTAACTGTGGACGCTGACACTCCCTT

CTTAATAGAGCAGTAAAGATAGATAGACTAAATATTAGTTATATAGCTAGCCAACAAAGG

GCGCGGCATGCATGTACGTATGTCCCAATAATATATATTATATACACTTCATTCAATAAG

GTCGCACGTCGTCCGTCGGTGGCGATACGCGTCACACCTCAGCGCGTGCCAGCGAGCGCA

TGCAGCTCGTCGTATGTACTGTACGTGCACCTACTCATGCACGCCATGTCGCTCATCGGC

GAAATTTTGCATCACACGGGCACCACGGTGGCGCGCCCCATCCCCATCTCCTCGTCCAGC

ACGTCCTCCTCCGTCGCCACCGGCGGCCCCGCCTTGGGGTCGAACCTCTTGCTCTTGGGG

TTCGTGGCCGGGTCGTACCTCTTGATCACCAGCACGGACGCCGACGTCCTGAACTCGGGC

GACACCAGGTAGCTCCCGACGGGGCCCAGCTCGTCGGGCCTCTCCACCAGCGGCGCCACC

GGCGGCATCCGCCCCACCACGAACACGTTGAACCCGGACAGCGACTCGATGGTCTCCAGC

ACCTGCTCCCTGGTGCACGCCTCCGGCTCCTCGAACCGCACCGACCCGTCCTTCACGGCC

CCGACCCTGGCCTTGAACGCCTCCACCGCGGCCTCGTCGGCCGCGCTCTCGGCCTCGTCC

AGCAGCGGCGGTCGGCTCGGCTGCAACCTGGCCAGCGTCACGGCGACGCCCGGGTGCTCG

GCCATGCGCGTCGCGTAGGCCAGCGCCTCGCGGTCGTCCGGCCCGCCGAAGAAGAGCACG

GCGACGGAGAAGGACACGTTCTTGGCCGACACCTGGGCGTGCCCGCCGAGGCCGCGGTCC

ACGAGGATGGCGACCGAGCAGGGCGCCTCGCGGAGCACGCGCTTGTTGACGGCGTGGTAC

GCCGACCCCAGCGACGCGAAGGAGCCGTCGTGGCCGAGCGCCCGGTGGTACGGCATCACC

ACGATGGCGGCGCGCTTGTCGGCGGCGCTGTCGATGACGTCGCGGTGGATGGTGTCCAGG

TCCGAGATGGCCGTCATGGCGCGCACCCGCACGGAGCTCAGCTGCTGGAACGCCTCGAAC

GCCACCACCACCTGCTCCGCCCGCTGCTCCTTGCCGCCGCTGAAAAAGGGCATGCCGTCG

CGGCGCGCGCGCTGTACCAGGCTGATGGCCGACGACCGCTCCGAGAGCTCCACCAGGTGC

ATTGCGTACATGGTGAGGCGGCGGCGCGCGGTGCCCCTGGACGCCTCCACCAGGTTCAGC

AGCGTGGGGATGTGGCGGTTGGTGTGGAAGCAGGCCAACACGCGCAGCTCGCCGTCCGCG

TCGCCCGCCGCGCACTCCACCGTGCGGCGCTTGTAGGGCACCGCCCGCCGCGCCGGCTTG

TAGATGGCCATCACGATGGGCGTCGTGATGAAGGTGGTGAAGAGCGCCATGAGCACCAGG

ATGGCGAACGCCTCGTCGTTCAGCACCTTGCGGTCGCGGCCGATGTTGAGGACGATGAGC

TCCACGAGCCCCTTGGTGTTCATGAGGAAGCCGAGCGTGACGGCCTCGCGGACGGGGATC

TTGACGAGCAGGGACGTGATCACCGTGCCGCCGATCTTGCCGAGGCACGCGTTAGCGATG

ACGAGCACGAGCAGGCCCCAGGACTTGGCGCCCTTGATGGTGGCCACGTCGGTCTTGAGC

CCGCTGGAGACGAAGTAGAGCGGGAGGAGGAGCCCCGAGATGAGGTCCTCCACCTTCTCG

ATGAGGACGCCGGCGAAAGGCCCGTCCTTGGGGACCACGATCCCCACCATGAAGGCGCCG

AACAGGGCATGGATGCCGATGGTGTCGGTGACGAAGCCGGCGGCGAGCACGATGGCGAGG

GTGGCGCAGATGTAGACCTCCTTGACCGGCTCGCCCTCGGGGGAGCGGCGCGCCATCCAG

GCCAGCACCGGGCGAAGGAAGAGCGAGACAGCAACGACAAATCCAGCGGCGGTGAGCAGC

ACCCACAGCGAGACGATGGGCGAGCCGGAGCCCGAGAGGGCGATGGCGAGCGCCAGGAGG

ATCCAGGCGGCGACGTCGTTGACCGCTGCGGCGGACATGGCCATGCGGCCGAGGTCCGTG

GTCAGCAGCTTGAGCTCGGCGAGGATGCGGGCCAGCACCGGGAACGCGGTGATGGAGAGC

GCCACGCCCATGAAGACGAGGAAGGGCCCCTGCGGCGTGCCCTTGACGATGGTGGCGCGG

AACGCGAACGACGTGCCGATGCCGAGCGCGAACGGCGCGCTGATGCCGGCCAGCGCGATG

GCCAGGGCCTTCTTGCCGGTGCGGCGGATGGCGGAGATGTCCAGCTCGAGGCCCACGAGG

AAGAGGAAGAAGAGCAGGCCGATGTTGGCCAGCGTGTCCAGCACCGTCATGCTCTGGGCC

GGGAACACGGCGTGCAGGAACCTGTGGCTCCGCCCCAGCGCCGACGGGCCTAGCAGGATT

CCTCCCTGCATCATACATGATCCATTCCAGGCAAAGTCGTCAGAACATGTAAAAAAAAAC

TAGAGTAGTATATCTTGCACGTACATATTAAGTCCATAACAAAAAGACGAAATTATCATT

TGACAACGAATTTAAAACTAATCATGGTATTAAAATAAATATCGTTAGTAGACTAAGTAT

AAAATGTATTTCTATGGTAACCATATTAGAAGATATATAAAAATCATGGTATCATTAGTT

TGCTCAAATTTATTTAAGAAAAAAAGGACTTGTCCTAAGTCTAGAACTGTACGTGACTAA

CTAGTTTCCTAATCAATAAGGTATCAAGCTGACGATGGTAGAAAGAAAAGAAGCAGGAAA

CAACTGCCTGCCCCAGTGATTCTTTTTCCTGATCACGCAATCTCAGCGGGTCAATGGTAG

AGGTTGGCAGAGGTGGACATGCACGCATGTATTACTCCATGCGTGAAGCAAGTCTACCAC

ATTTCCTCGCGCACAATAATGCACATTAAAAAAAACAGCCCGGCCGCGTGTCCTGTTTTT

GTGTACACAGCTATTGCTGCTGTGGTGTGGATACTACCCTCCTCCGTGTATACGTGTATA

GACTGGCGTTGACTTTGGTGACTAGAATAGAAAGAAACAAGACAGTATAGAAGCGAAGGT

CACTTGTTGCTGAGATAGATAGACGATGAACCATGCCTAGAAACCAGCCCTCGTTCACTT

ATTTGACCAGTTCAGTGTGTGTATGTACAACATGTTGAAATTAGGCTCACTAATGTGTGG

CTCAATTCAAAAGGAAGTCAAGGTGATGTGCAGAGACAATATAGGATTTTAGTCTCATAT

TGTTCATCTAGATAGGAGAGAACGAACTTATATGTGGGAGTGTTCCTGACTCTTCACATG

AGACAAATTCCCAAGTGTGGTTCATGGAATTTAGCCGATCAAACATGTCAAGTTGTTGCG

ACCCTCTATAAAGGGGAGGGGTCTTTTAGCCGAAGGGCACACGACACAGCAACCGACTCA

CCTTCTCATTCTCTTCTCTCCGTGTCGTCGCTTCTCTACCCTGCCGTGCAGCAGATCAAG

GGAGGGCGGTGTCTTCGAACCAACGTCGGTGTTTTGCCGACCTGCACGGGTGCCGGCGAA

TAAGGTTTCTGGGAAGTGCTCCACGCACGTTCGATCGGGTCCCTTGTTATCACTCGTGCA

AATTGATATAAGTAAATCTGCATTTTTTCTTTATTGTGACGACGATCTTATGGTTAGATC

TGTTGTCGATTATTTCTTTTATACATTAGTGTTAGTTCAGATCTATTTATATTCTAGTGT

TATGGATACTACCTGCCAGTAACTCTGTGTTTTACGTTTTACGCGTGTTATCAAATTAAT

CATAGACCATGTTAATTTTATATAACACAACATATGAGTCGTCGTGTGGACGTGTGGGGA

ACTACGTACGTGTCAGATAGTCAAAAGGAGCAAGGCCACTGAGTAGTCGATGTCAGGCGC

GTGTGGCGAGCTAGCTAATTAAGGGGTTTATAACGCAACATAACTGGGATTTGGGAGTTT

GATGGCCGGAGGTGTCGATCCGATCTCTGAGGAGGAGAATCCGCGCAGCAGCAGCAGAAG

GGGGTATGTGAGATCCGGCCGGCCAGCCTATCCTGATTCGGTGATTCCTGAGGATCCGGG

CCGGATATGCATATGCTTCCCCCGCGCCCTTGCCGTTGCCGTTGCCGTTGCCGATGATCC

ATCAATTCGGCGTACGCATGGCATCATGCAGTCTGCATGTGTGTCCATGTACCAAGTACA

GCTGCTAGTGCTACCTCACCAAGCTCTAGGGATGGCGAGACCAAGAAAATTCTACCGGTA

GTGCCGTCACGACGTACGCGCGCGTGCCGCAGTTCTTGCAACCGTTATACCAACTAAAAC

ATAACAACGGCGTGGACACGACCGTCCGGCCGGCGTGCCGCGGCGTGCCGCGTAGGTAGT

ATAGTAGGAACCCACGAAGTCAGCCTGGACGCGGGACTCCGGCGGGACATCGCTGTTCGT

TGTGAACTTCGTTACTCCAAAGAGGGCGGGGGACTAGTCCACCACCACTGCCCAACCGAT

CCAGCCGGGCGGGCGCGCGCGGGAGGAGGGTCCTCCCGTCCGCCCTCCGCGAGGACGAGG

ACGAGGACGAGGACAACAACGACCGGCCGCGAGAAAAAATGGAACAGGAGAGCGAGAGTG

GTGGATGGAATAGCCGGGGCGGCGGAGCCGGGCGGAGCGCGCGGGTGGGAACATGGATTG

GAACGGGGAAAGAGCCGCGCCGCCGCCGCCGCCGCCAGCCGGAGATTCATGCATGCATGC

ATGCATGCATGCGCGCATGTATAACGAGGGAGCGAGCCGGGGGGAAAGGGTAGGGTACTC

ACGATGATCTCGGCGATGACGCGGGGCTGGCGCAGCGGGCGGAGCAGGTAGGCGAGGCCG

CGGGTGACGACGACGACCAGGCAGATCTGCAGGATGGCCAGCGGCAGCGCGTAGTCCAGC

GGGTTCTCGCCCTGGAACGCCCCCTGCGACGTCGCCTTCATCGGCCCCGGGCACATGGCG

GCCGCCGCCATCTGTATATCTCCGACGACGACGACCGATCGGTCCGCCGCCGCGTGGGCC

GGTGCTGCTGCCGCCTTAGCTAAGAGAGTTACTACCTAGCTACTACCACCAGCGATCCGC

CGCCGCCGCGCGCGTGGGTACGCTGGACGATTGAGACGACTGGATCGAATTGACTTGGTA

GCAGCAAATAAACGAAACAGAGATCGATATCCGCGATACAAGCAGTTGGTTGTGCGTGCT

GGGTCGGCGGGGGGGTTTTATAGATGAGATGAGATGATATAGGGCGAGACGGGGATGAAG

GCAGCCGGCCCCCACGCCACGCCACGTCACCGTCTACGTGTCCGCGGCCGTTGCGGCAGG

CAGGCACAGGCCGAGAGCCCGAGAGACCCGGACGACGGAGGATTGGCATAATGCCGATCC

CTGCTTTTGTTTAGGGCAATAATGCAACGGGGGGAGGCGTTGACATTGCGTAATTGCACA

ATTGCACTTGCACCCCACGCGCCGCCCAAACGGCTCGGTCCTAGCTAGCTAGCTATGAGT

ACACAGATTGTTAGGAATCTAGCCGATATTCCGTGCTCAGTTTAAACAAAAAATAACACG

GTAAATTTAGGACGGCATGTATATGCATACTTATAATACTACCTCAGTAATTTAGGTTCT

ATTTGGAAGTAAGACAATTTTTAAGAAACTGTTTTTTATATTTTAGCTAGGAGAAGGTGA

CTTCTTGGTTTTTTAAGAAACCGGGAATCCAGTTTCTATAAACTAACGCATAAACTGATA

TGTTTAGAACTACACCAATTTCCATAAGCCAATTTATTAAAAACTGGGTGCTTCCAAAAA

TGCCCTTATATATACGACTACCGACCACGAAAGAAAAAATAGTACTGAAGAATTTAACAA

GTTCATATAGTAACATATCCTTAGCAGAGGGACCCAATCTTTAC

>*pgl-sd_gy598*, sequence of the PCR amplicon from *pgl-sd* with gy598

TTCCCCGTTCCAATCCATGTTCCCACCCGCGCGCTCCGCCCGGCTCCGCCGCCCCGGCTA

TTCCATCCACCACTCTCGCTCTCCTGTTCCATTTTTTCTCGCGGCCGGTCGTTGTTGTCC

TCGTCCTCGTCCTCGTCCTCGCGGAGGGCGGACGGGAGGACCCTCCTCCCGCGCGCGCCC

GCCCGGCTGGATCGGTTGGGCAGTGGTGGTGGACTAGTCCCCCGCCCTCTTTGGAGTAAC

GAAGTTCACAACGAACAGCGATGTCCCGCCGGAGTCCCGCGTCCAGGCTGACTTCGTGGG

TTCCTACTATACTACCTACGCGGCACGCCGCGGCACGCCGGCCGGACGGTCGTGTCCACG

CCGTTGTTATGTTTTAGTTGGTATAACGGTTGCAAGAACTGCGGCACGCGCGCGTACGTC

GTGACGGCACTACCGGTAGAATTTTCTTGGTCTCGCCATCCCTAGAGCTTGGTGAGGTAG

CACTAGCAGCTGTACTTGGTACATGGACACACATGCAGACTGCATGATGCCATGCGTACG

CCGAATTGATGGATCATCGGCAACGGCAACGGCAACGGCAAGGGCGCGGGGGAAGCATAT

GCATATCCGGCCCGGATCCTCAGGAATCACCGAATCAGGATAGGCTGGCCGGCCGGATCT

CACATACCCCCTTCTGCTGCTGCTGCGCGGATTCTCCTCCTCAGAGATCGGATCGACACC

TCCGGCCATCAAACTCCCAAATCCCAGTTATGTTGCGTTATAAACCCCTTAATTAGCTAG

CTCGCCACACGCGCCTGACATCGACTACTCAGTGGCCTTGCTCCTTTTGACTATCTGACA

CGTACGTAGTTCCCCACACGTCCACACGACGACTCATATGTTGTGTTATATAAAATTAAC

ATGGTCTATGATTAATTTGATAACACGCGTAAAACGTAAAACACAGAGTTACTGGCAGGT

AGTATCCATAACACTAGAATATAAATAGATCTGAACTAACACTAATGTATAAAAGAAATA

ATCGACAACAGATCTAACCATAAGATCGTCGTCACAATAAAGAAAAAATGCAGATTTACT

TATATCAATTTGCACGAGTGATAACAAGGGACCCGATCGAACGTGCGTGGAGCACTTCCC

AGAAACCTTATTCGCCGGCACCCGTGCAGGTCGGCAAAACACCGACGTTGGTTCGAAGAC

ACCGCCCTCCCTTGATCTGCTGCACGGCAGGGTAGAGAAGCGACGACACGGAGAGAAGAG

AATGAGAAGGTGAGTCGGTTGCTGTGTCGTGTGCCCTTCGGCTAAAAGACCCCTCCCCTT

TATAGAGGGTCGCAACAACTTGACATGTTTGATCGGCTAAATTCCATGAACCACACTTGG

GAATTTGTCTCATGTGAAGAGTCAGGAACACTCCCACATATAAGTTCGTTCTCTCCTATC

TAGATGAACAATATGAGACTAAAATCCTATATTGTCTCTGCACATCACCTTGACTTCCTT

TTGAATTGAGCCACACATTAGTGAGCCTAATTTCAACATGTTGTACATACACACACTGAA

CTGGTCAAATAAGTGAACGAGGGCTGGTTTCTAGGCATGGTTCATCGTCTATCTATCTCA

GCAACAAGTGACCTTCGCTTCTATACTGTCTTGTTTCTTTCTATTCTAGTCACCAAAGTC

AACGCCAGTCTATACACGTATACACGGAGGAGGGTAGTATCCACACCACAGCAGCAATAG

CTGTGTACACAAAAACAGGACACGCGGCCGGGCTGTTTTTTTTAATGTGCATTATTGTGC

GCGAGGAAATGTGGTAGACTTGCTTCACGCATGGAGTAATACATGCGTGCATGTCCACCT

CTGCCAACCTCTACCATTGACCCGCTGAGATTGCGTGATCAGGAAAAAGAATCACTGGGG

CAGGCAGTTGTTTCCTGCTTCTTTTCTTTCTACCATCGTCAGCTTGATACCTTATTGATT

AGGAAACTAGTTAGTCACGTACAGTTCTAGACTTAGGACAAGTCCTTTTTTTCTTAAATA

AATTTGAGCAAACTAATGATACCATGATTTTTATATATCTTCTAATATGGTTACCATAGA

AATACATTTTATACTTAGTCTACTAACGATATTTATTTTAATACCATGATTAGTTTTAAA

TTCGTTGTCAAATGATAATTTCGTCTTTTTGTTATGGACTTAATATGTACGTGCAAGATA

TACTACTCTAGTTTTTTTTTACATGTTCTGACGACTTTGCCTGGAATGGATCATGTATGA

TGCAGGGAGGAATCCTGCTAGGCCCGTCGGCGCTGGGGCGGAGCCACAGGTTCCTGCACG

CCGTGTTCCCGGCCCAGAGCATGACGGTGCTGGACACGCTGGCCAACATCGGCCTGCTCT

TCTTCCTCTTCCTCGTGGGCCTCGAGCTGGACATCTCCGCCATCCGCCGCACCGGCAAGA

AGGCCCTGGCCATCGCGCTGGCCGGCATCAGCGCGCCGTTCGCGCTCGGCATCGGCACGT

CGTTCGCGTTCCGCGCCACCATCGTCAAGGGCACGCCGCAGGGGCCCTTCCTCGTCTTCA

TGGGCGTGGCGCTCTCCATCACCGCGTTCCCGGTGCTGGCCCGCATCCTCGCCGAGCTCA

AGCTGCTGACCACGGACCTCGGCCGCATGGCCATGTCCGCCGCAGCGGTCAACGACGTCG

CCGCCTGGATCCTCCTGGCGCTCGCCATCGCCCTCTCGGGCTCCGGCTCGCCCATCGTCT

CGCTGTGGGTGCTGCTCACCGCCGCTGGATTTGTCGTTGCTGTCTCGCTCTTCCTTCGCC

CGGTGCTGGCCTGGATGGCGCGCCGCTCCCCCGAGGGCGAGCCGGTCAAGGAGGTCTACA

TCTGCGCCACCCTCGCCATCGTGCTCGCCGCCGGCTTCGTCACCGACACCATCGGCATCC

ATGCCCTGTTCGGCGCCTTCATGGTGGGGATCGTGGTCCCCAAGGACGGGCCTTTCGCCG

GCGTCCTCATCGAGAAGGTGGAGGACCTCATCTCGGGGCTCCTCCTCCCGCTCTACTTCG

TCTCCAGCGGGCTCAAGACCGACGTGGCCACCATCAAGGGCGCCAAGTCCTGGGGCCTGC

TCGTGCTCGTCATCGCTAACGCGTGCCTCGGCAAGATCGGCGGCACGGTGATCACGTCCC

TGCTCGTCAAGATCCCCGTCCGCGAGGCCGTCACGCTCGGCTTCCTCATGAACACCAAGG

GGCTCGTGGAGCTCATCGTCCTCAACATCGGCCGCGACCGCAAGGTGCTGAACGACGAGG

CGTTCGCCATCCTGGTGCTCATGGCGCTCTTCACCACCTTCATCACGACGCCCATCGTGA

TGGCCATCTACAAGCCGGCGCGGCGGGCGGTGCCCTACAAGCGCCGCACGGTGGAGTGCG

CGGCGGGCGACGCGGACGGCGAGCTGCGCGTGTTGGCCTGCTTCCACACCAACCGCCACA

TCCCCACGCTGCTGAACCTGGTGGAGGCGTCCAGGGGCACCGCGCGCCGCCGCCTCACCA

TGTACGCAATGCACCTGGTGGAGCTCTCGGAGCGGTCGTCGGCCATCAGCCTGGTACAGC

GCGCGCGCCGCGACGGCATGCCCTTTTTCAGCGGCGGCAAGGAGCAGCGGGCGGAGCAGG

TGGTGGTGGCGTTCGAGGCGTTCCAGCAGCTGAGCTCCGTGCGGGTGCGCGCCATGACGG

CCATCTCGGACCTGGACACCATCCACCGCGACGTCATCGACAGCGCCGCCGACAAGCGCG

CCGCCATCGTGGTGATGCCGTACCACCGGGCGCTCGGCCACGACGGCTCCTTCGCGTCGC

TGGGGTCGGCGTACCACGCCGTCAACAAGCGCGTGCTCCGCGAGGCGCCCTGCTCGGTCG

CCATCCTCGTGGACCGCGGCCTCGGCGGGCACGCCCAGGTGTCGGCCAAGAACGTGTCCT

TCTCCGTCGCCGTGCTCTTCTTCGGCGGGCCGGACGACCGCGAGGCGCTGGCCTACGCGA

CGCGCATGGCCGAGCACCCGGGCGTCGCCGTGACGCTGGCCAGGTTGCAGCCGAGCCGAC

CGCCGCTGCTGGACGAGGCCGAGAGCGCGGCCGACGAGGCCGCGGTGGAGGCGTTCAAGG

CCAGGGTCGGGGCCGTGAAGGACGGGTCGGTGCGGTTCGAGGAGCCGGAGGCGTGCACCA

GGGAGCAGGTGCTGGAGACCATCGAGTCGCTGTCCGGGTTCAACGTGTTCGTGGTGGGGC

GGATGCCGCCGGTGGCGCCGCTGGTGGAGAGGCCCGACGAGCTGGGCCCCGTCGGGAGCT

ACCTGGTGTCGCCCGAGTTCAGGACGTCGGCGTCCGTGCTGGTGATCAAGAGGTACGACC

CGGCCACGAACCCCAAGAGCAAGAGGTTCGACCCCAAGGCGGGGCCGCCGGTGGCGACGG

AGGAGGACGTGCTGGACGAGGAGATGGGGATGGGGCGCGCCACCGTGGTGCCCGTGTGAT

GCAAAATTTCGCCGATGAGCGACATGGCGTGCATGAGTAGGTGCACGTACAGTACATACG

ACGAGCTGCATGCGCTCGCTGGCACGCGCTGAGGTGTGACGCGTATCGCCACCGACGGAC

GACGTGCGACCTTATTGAATGAAGTGTATATAATATATATTATTGGGACATACGTACATG

CATGCCGCGCCCTTTGTTGGCTAGCTATATAACTAATATTTAGTCTATCTATCTTTACTG

CTCTATTAAGAAGGGAGTGTCAGCGTCCACAGTTAACGTTATGTTTTGATTATTGTATTT

CTAGAATTCGTATCGAGTTATTTTTGGACTACATATTTTTTGAATGTTTAAATTATATCT

TCTTGTGTCATATTAAACATCAATTATATTTTTTGTTTGGTTTAAAATATTTTATAGCCT

TAGCTTAGTAACCCCTCTTGGATCAATCCTAGATCCGTCACTGGATGTCATGGACTATGG

TGTGACGACCAATCTCTCAGCGGATGATGAGTGCATGCACCAGCAGGATTAGGCCCCACA

TATCAAGTTTTAGATCTTTGTGATGGAAGTGGAAATGTACCACATAGTCCATAATTGTCC

GATCGACGGTGCTGGACATCTGACAAGGGCAAATAATGCTCTACACTTGATGCTCTCTCT

CTCTCCCCCCTCTCTCTCTCTCTGGTAGTTGGTTTAACGCTAACCAACAAGAAACCGCAT

AAGGGAAAGGGAGATCAAATCCCCAATTATTGCTGGCATCCGTGAACTAAGCAAGCGTCC

ACACTAACACACAGGCACTGTCTATTTTCTACGATCTCTACTTGAAATGTTAGTCTAGCA

CGTAGAAGCATCTTCCTTCTTCCATAAACTTGTAAATGAATGTTGCCTGTGGGGGTGTGG

TTTGGTTGCAATGAATTGGAGCGGATCCATGGAAAAAAAATCATTTGTTTAATTTTAAAT

AGCAAGGAATTTTCTCTACCATGCGCTACCGGAATCCGGCTCTTTACCGAGTTATTTATT

TTTGACACTCGACAAAGAGTTTCTTTGCCGCCGAGTGTTTTTTTTCAACACTACGCAAAG

ATAATTTAAAAATCGTATTTTAAAGCAGTAAATTAATTCAAATGATAGGACGATAAATAG

GGTTTTTTCGCCGAGGTTGCGAGTTCAAATCTTACCAGTCACAAAACACCTGAATTCAGT

TAAAAAAATGGTGAAAAAATGATAGGACGATGGGTAGGGCAATGGTGATGGTTAGGGTGT

TGTTCTCCTAATCCCTAATTTAGAAAATTGTTTTGATGTTTTTTTTTCGATGTTAATTTG

CCGAGTGTTTTTTGACACTCGACAAAGTATTTGCTGAGTGCCCCCAAAAAATACTCGGTA

AAGAACTATTTGTCGATAAAATATCTGTCGAGCGTTATTTGCCGAGTGTTTGAGACACTC

GGTGAAGAATGCAAGGTCGATAGTGCTAATTCTCTTGTCACCCAACGTTCTCCGCCAAAG

CCTTCGACATGCGGCGGATATCTTACATCGTATGTGTGTGTGAGGCTACTCAATGATGCG

GCGGATATCATGCATACAAATAAAACATTATTCGGTATAGTTTGCGGCGTGCATGGCAA
