# Supplementary material for: A large deletion conferring pale green leaves of maize
Source: BMC Plant Biol. 2023 Jul 14;23:360. doi: 10.1186/s12870-023-04360-2 (PMC10347855; doi:10.1186/s12870-023-04360-2)
Supplement: Supplementary file 2 — Additional file 2. [file 12870_2023_4360_MOESM2_ESM.doc]

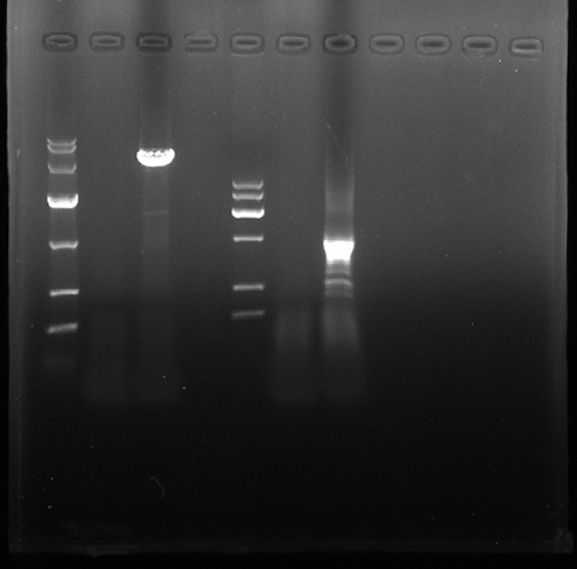

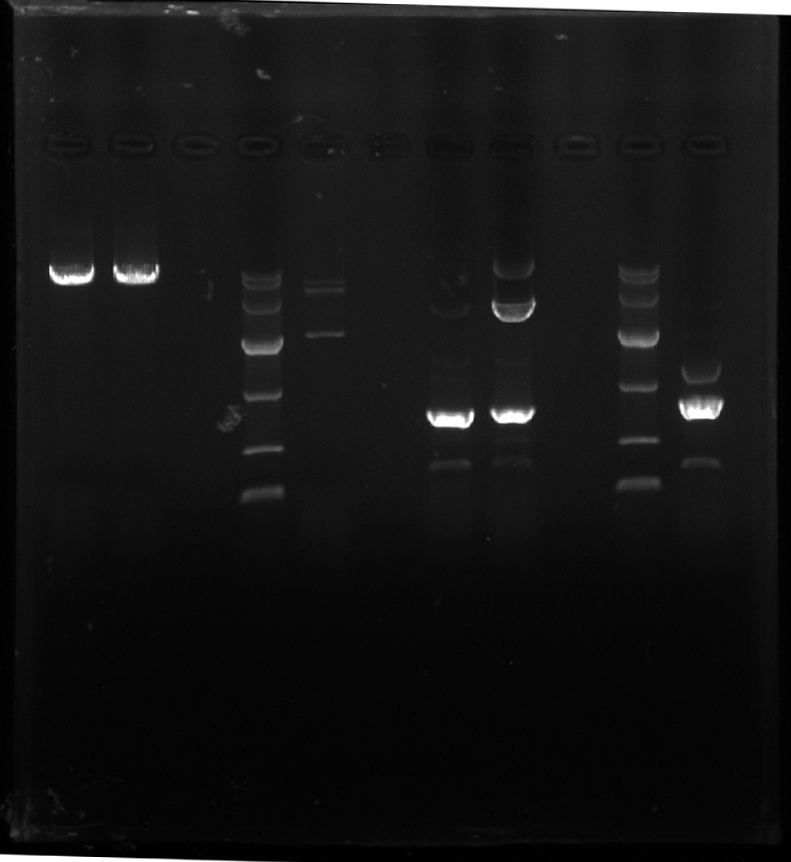


Supplementary file1. The structure variation in *pgl-sd*. Triangles indicated expected PCR products. a, the original gel of the amplification of *Zm00001eb031870* with the primer pair gy573. Lane 1, 5: molecular weight markers; Lane 2, 3: genomic DNA of *pgl-sd* and Q319 was used, respectively; Lane 6, 7, cDNA of *pgl-sd* and Q319 was used, respectively; other lanes were not loaded with samples. b, the original gel of amplificons with gy598 (lane 1, 2, 5) and gy599 (lane 7, 8, 11), respectively. Lane 4 and 10: molecular weight markers; Lane 1, 2, 7, 8: genomic DNA of *pgl-sd* was used; Lane 5, 11: genomic DNA of Q319 was used; other lanes were empty.

1 2 3 4 5 6 7 8 9 10 11

1 2 3 4 5 6 7 8 9 10 11

7.0Kb

4.0Kb

1.5Kb

1.0Kb

a

b

7.0Kb

2.0Kb
