# Supplementary material for: A large deletion conferring pale green leaves of maize
Source: BMC Plant Biol. 2023 Jul 14;23:360. doi: 10.1186/s12870-023-04360-2 (PMC10347855; doi:10.1186/s12870-023-04360-2)

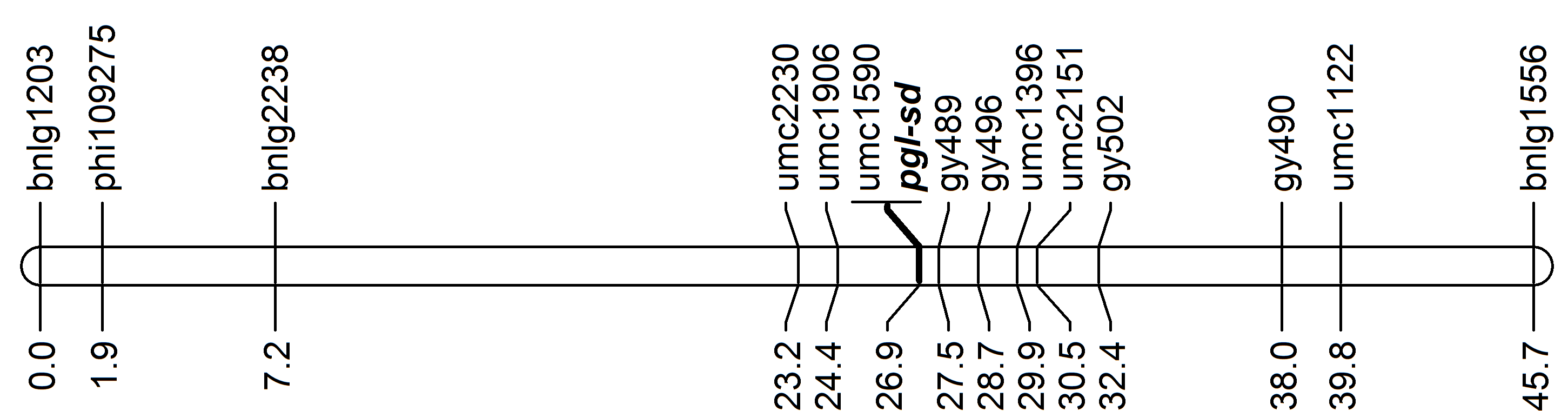

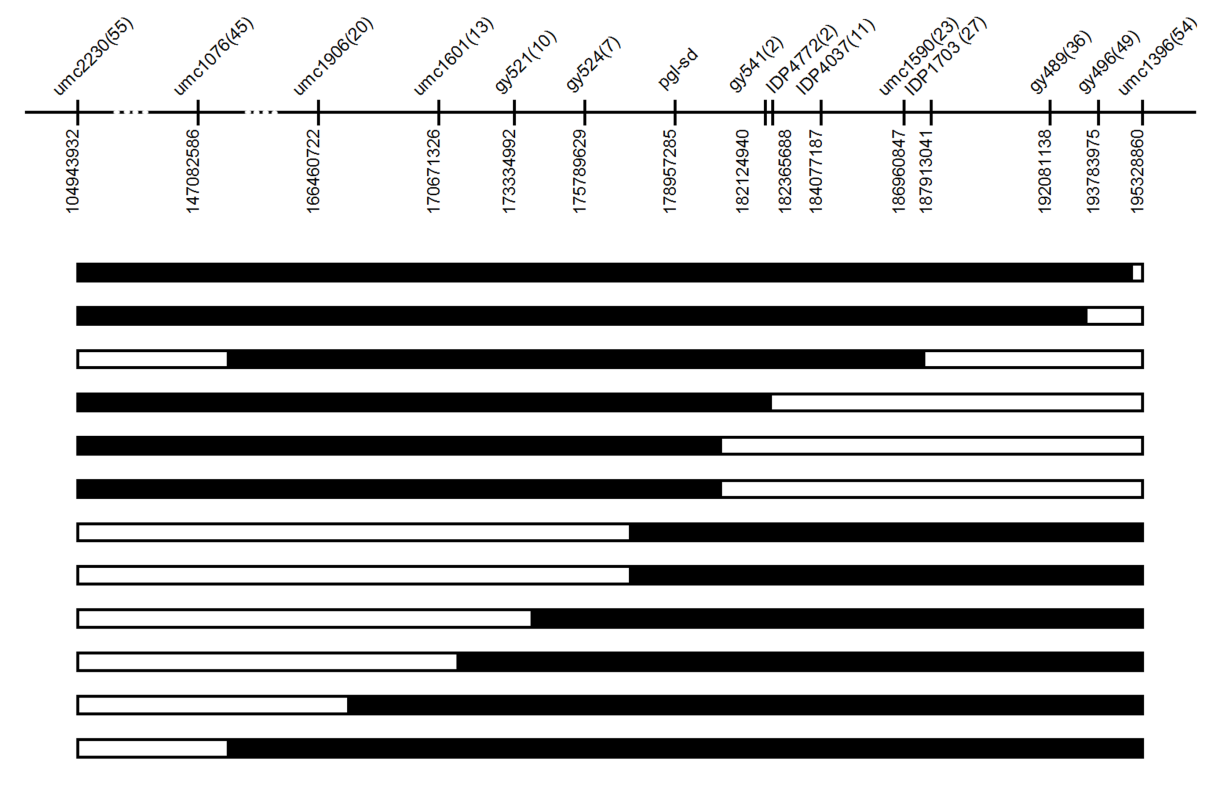


a

b

c

Fig. S1. Fine mapping of *pgl-sd* with Zheng58/*pgl-sd* F_2_ population. a, linkage mapping of *pgl-sd* with the Zheng58/*pgl-sd* F_2_ population (units = cM). b, physical coordinates of markers on the chromosome 1 for fine mapping of *pgl-sd* with Zheng58/*pgl-sd* F_2_ population based on RefGen_v5 (units = bp). The number of recombinants between each marker and *pgl-sd* were showed in bracket following the marker. c, genotype of representative recombinants, with black indicating *pgl-sd* genotype and white indicating heterozygous genotype. Codes of these plants from top to below were b6.03.03, b5.54.03, b5.02.08, b2.02.15, b3.167, b6.81.07, b5.64.06, b5.67.01, b6.45.04, b6.94.13, b1.07.01, and b6.18.17, respectively.


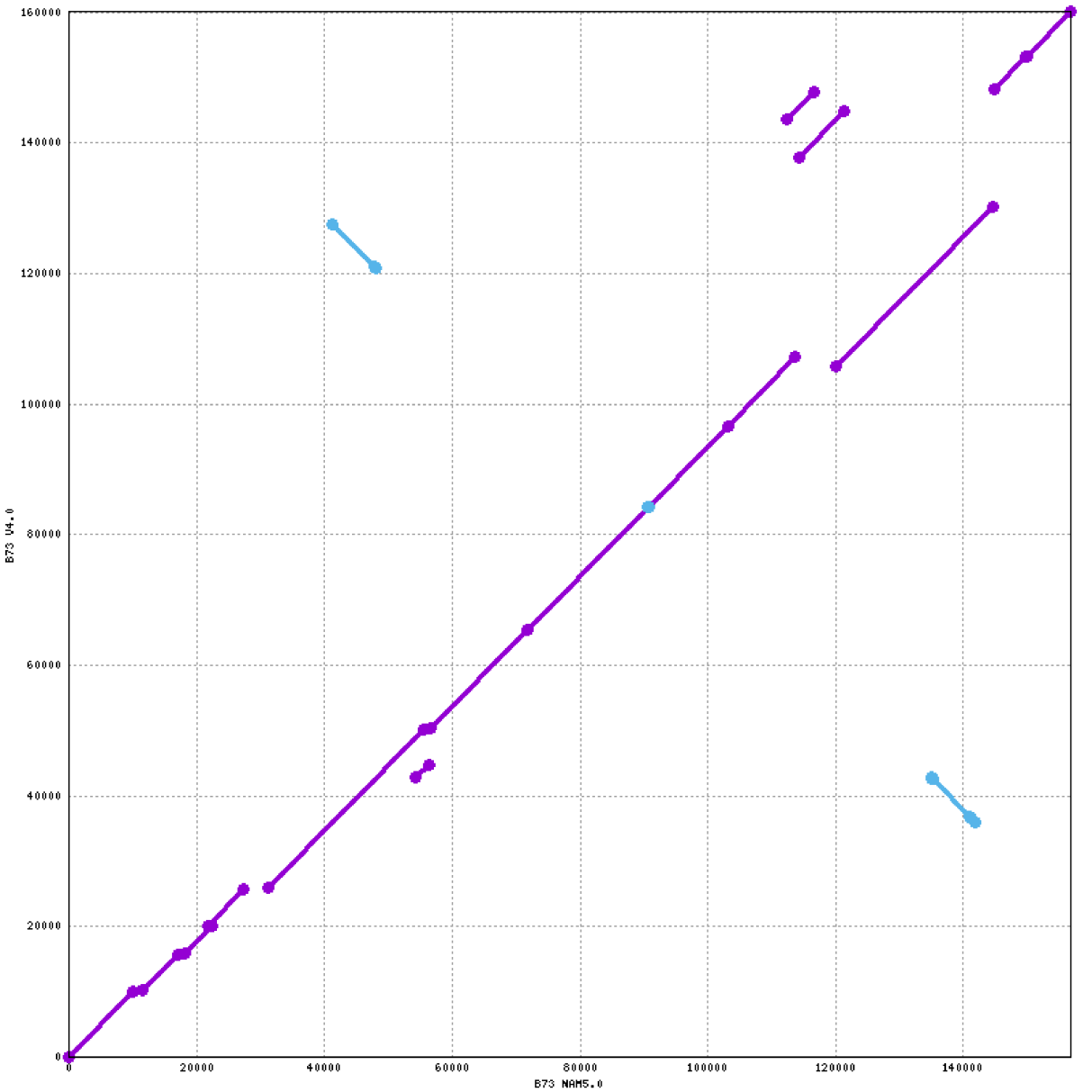


Fig. S2. Plot of the sequence alignment of the target interval bracketed by gy546 and gy548 of RefGen_v3 to the corresponding region of RefGen_v5 with Mumer.

Fig. S3. RNA-Seq expression of 7 genes in the target region delimited by gy546 and gy548. (https://www/maizgdb.org). 1. 6-7_Internode, 2. 7-8_Internode, 3. Meristem_16-19_Day, 4. Ear_Primordium_2-4mm, 5. Ear_Primordium_6-8mm, 6. Embryo_20DAP, 7. Embryo_38DAP, 8. Endosperm_12DAP, 9. Endosperm_Crown_27DAP, 10. Germinatin_Kernels_2DAI, 11. Pericarp/Aleurone_27DAP, 12. Leaf_Zone_1_Symmetrical, 13. Leaf_Zone_2_Stomatal, 14. Leaf_Zone_3_Growth, 15. Matrue_Leaf_8, 16. Primary_Root_5_Days, 17. Root_Cortex_5_Days, 18 Root_Elongation_Zone_5_Days, 19. Root_Meristem_Zone_5_Days, 20. Secondary_Root_7-8_Days, 21. B73_Mature_Pollen, 22. Female_Spikelet, 23. Silk.


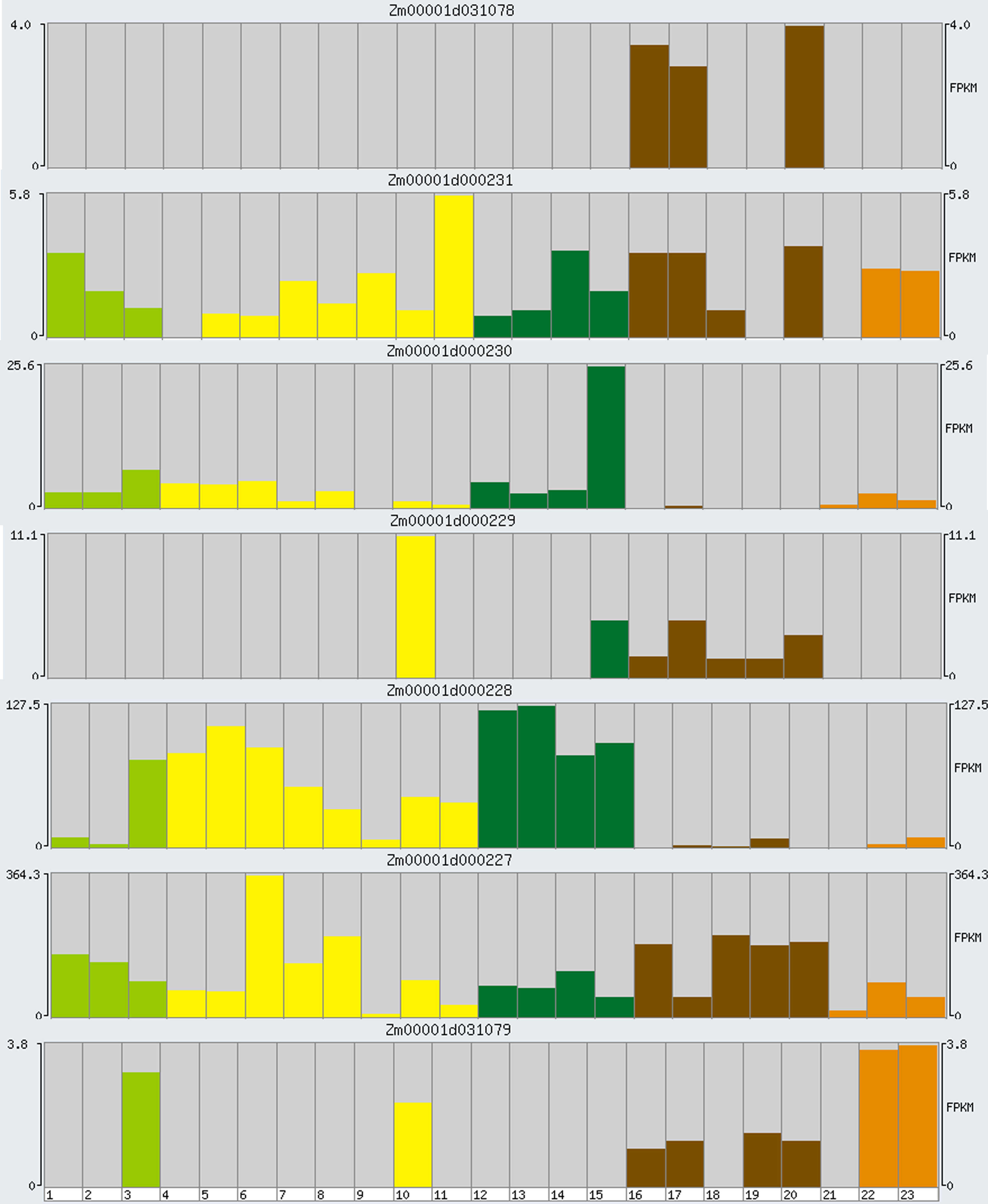

Supplement: Supplementary file 3 — Additional file 3. [file 12870_2023_4360_MOESM3_ESM.doc]
